# The role of the tumor microenvironment in mediating radiopharmaceutical therapy: bridging nuclear medicine and cancer immunotherapy

**DOI:** 10.1016/j.mmr.2026.100038

**Published:** 2026-05-19

**Authors:** Zhi-Peng Wang, Ai-Qing Li, Dilinaer Wusiman, Rui-Cheng Wu, Ji-Min Zhu, Xin-Rui Li, Yuan-Ning Guo, Premkamon Chaipanichkul, Uzoamaka Adaobi Okoli, Siang Boon Koh, Jie Wang, Deng-Xiong Li, Xiao-Dong Jin, Da-Hong Zhang, Cheema Umber, Qi Zhang, De-Chao Feng

**Affiliations:** a Department of Urology, Sichuan Provincial People’s Hospital, University of Electronic Science and Technology of China, Chengdu 610072, China; b The Center of Psychosomatic Medicine, Sichuan Provincial People’s Hospital, University of Electronic Science and Technology of China, Chengdu 610072, China; c Purdue Institute for Cancer Research, Purdue University, West Lafayette, IN 47907, USA; d Division of Surgery & Interventional Science, University College London, London W1W 7TS, UK; e Department of Urology, Institute of Urology, West China Hospital, Sichuan University, Chengdu 610041, China; f Department of Gastroenterology and Hepatology and Shanghai Institute of Liver Diseases, Zhongshan Hospital, Fudan University, Shanghai 200032, China; g Department of Rehabilitation, the Affiliated Hospital of Southwest Medical University, Luzhou 646000, Sichuan, China; h Rappaport Family Institute for Research in the Medical Sciences, Technion-Israel Institute of Technology, Haifa 31096, Israel; i Department of Radiology, Division of Radiation Oncology, Faculty of Medicine, Khon Kaen University, Khon Kaen 40002, Thailand; j Basic and Translational Cancer Research Group, Department of Pharmacology and Therapeutics, College of Medicine, University of Nigeria, Enugu 340001, Nigeria; k Faculty of Health and Life Sciences, University of Bristol, Bristol BS8 1TD, UK; l Department of Urology, the First Affiliated Hospital of Zhejiang Chinese Medical University (Zhejiang Provincial Hospital of Chinese Medicine), Hangzhou 310009, China; m Urology & Nephrology Center, Department of Urology, Zhejiang Provincial People’s Hospital (Affiliated People’s Hospital), Hangzhou Medical College, Hangzhou 310014, China

**Keywords:** Radiopharmaceutical therapy (RPT), Tumor microenvironment (TME), Cancer immunotherapy, Theranostics, Nuclear medicine

## Abstract

Radiopharmaceutical therapy (RPT) is a pivotal modality in cancer treatment, with its efficacy substantially modulated by the tumor microenvironment (TME). The TME regulates RPT penetration and influences therapeutic outcomes. Concurrently, RPT actively reconfigures the TME by enhancing immune activation and partially reversing immunosuppression. Specifically, RPT induces tumor cell senescence and immunogenic cell death, and catalyzes immune activation. RPT diminishes immunosuppressive elements, including regulatory T cells and tumor-associated macrophages, and augments populations of CD8⁺ T cells and natural killer cells, thereby fostering a more immunostimulatory TME. Additionally, RPT modulates critical cytokines and upregulates immune checkpoint molecules, bolstering anti-tumor immunity. Nevertheless, pathological features like hypoxia and extracellular matrix stiffness within the TME can hinder RPT biodistribution and therapeutic efficacy. Strategically targeting the TME through approaches, such as fibroblast depletion, hypoxia mitigation, metabolic reprogramming, nerve-immune-cancer interactions, or nanocarrier drug delivery, can amplify RPT effectiveness. Combination immunotherapies that integrate RPT with immune checkpoint inhibitors, chimeric antigen receptor (CAR)-T cells, or cancer vaccines have demonstrated enhanced anti-tumor responses and survival advantages in malignancies such as prostate cancer and neuroendocrine tumors. Given the complexity and heterogeneity of the TME, emerging strategies, including radiation-responsive nanocarriers, CAR-T cells engineered to secrete radiosensitizers, senolytic therapies, and artificial intelligence-driven multimodal data integration, are promising in addressing the challenges and refining precision cancer therapy. In summary, the TME serves as a critical bridge between RPT and immunotherapy; elucidating the interplay among these three elements is essential for advancing combination strategies.

## Background

Cancer remains one of the primary causes of mortality worldwide [1], [2]. The steady rise in both the incidence and mortality rates of cancer in recent years, exacerbated by the global increase in the aging population, highlights substantial challenges to public health across the globe [3], [4], [5]. The International Agency for Research on Cancer reported approximately 19.3 million new cases of cancer and nearly 10 million cancer-related deaths globally in 2020 [6].

Radiotherapy (RT) is a cornerstone for half of the cancer patients, while immunotherapy (IT) offers durable remission for some advanced cases [7]. Limited efficacy of either alone drives interest in combinations. This combination has demonstrated significant clinical success. The strongest evidence comes from the phase III PACIFIC trial in patients with advanced non-small cell lung cancer (NSCLC). Patients receiving the programmed cell death ligand 1 (PD-L1) inhibitor durvalumab achieved a median progression-free survival of 16.8 months, vs. 5.6 months for the placebo group [8]. Moreover, durvalumab improved the overall survival in patients with unresectable NSCLC after chemotherapy plus concurrent RT [9]. Furthermore, a pooled analysis of two randomized trials showed statistically significant efficacy for RT combined with IT in advanced NSCLC [10], [11].

Radiopharmaceutical therapy (RPT) involves the delivery of radioactive atoms to cancer-associated targets and represents a promising strategy for cancer treatment. This therapeutic approach utilizes radioactive isotopes to specifically target and eradicate cancer cells, such as iodine-131 and radium-223 [12], [13]. The advantages of RPT lie in its high specificity and effectiveness, which minimize damage to normal tissues [13]. RPTs exert their therapeutic effects through multiple mechanisms, including direct radiation damage, induction of apoptosis, disruption of the cell cycle, and regulation of immune cells [14]. These mechanisms largely overlap with RT’s effects on the tumor microenvironment (TME). However, unlike RT’s high-dose, short-duration local irradiation, RPT’s continuous low-dose-rate exposure and tumor-selective biodistribution help avoid unnecessary irradiation of circulating immune cells, favoring combination with immune checkpoint inhibitors (ICIs) [15]. Regarding toxicity, RT toxicity depends on the anatomical irradiation field, while RPT toxicity relates to radioligand biodistribution. RPT’s systemic targeting and low-dose-rate features enable treatment of widely metastatic malignancies and may allow more specific modulation of systemic anti-tumor immune responses, with its toxicity mainly governed by pharmacokinetics [16].

To optimize the efficacy and safety of RPT, a series of innovative approaches is under development. These include the use of computational models to refine treatment strategies, such as multiscale modeling frameworks that integrate whole-body pharmacokinetics with tumor-scale biophysical models [17], an AI-based “computational nuclear medicine” paradigm [18], physiologically-based pharmacokinetic models guiding RPT dosing in prostate cancer [19], and spatiotemporal kinetic models based on clinical imaging for personalized RPT administration [20]. To enhance RPT delivery efficiency, novel delivery systems [e.g., pH-responsive charge-switching nanoparticles (NPs)] [21] and strategies aimed at reducing off-target toxicity (e.g., magnetothermal-controlled release nanoplatforms) [22] are also being developed. However, the successful implementation of these new strategies aimed at optimizing RPT largely depends on a profound understanding of TME.

The TME constitutes a multifaceted ecosystem comprised of cancer cells, stromal cells, immune cells, the extracellular matrix (ECM), and molecules associated with cellular components of the TME [23], [24], [25], [26], [27], [28]. Immune cells possess dual functions of either promoting or inhibiting tumors [29], [30], [31]. The ECM is a key biophysical facet of the TME that provides tissues with structural support [32]. Within the TME, ECM remodeling results in abnormal protein deposition, increased stiffness, and altered composition [33], which can promote tumor cell invasion, metastasis, and therapy resistance [34]. Since the tumors require a blood supply to sustain their growth, the TME actively promotes angiogenesis [35], [36]. Hypoxia, a key biophysical factor, supplying nutrients and oxygen to tumors [37]. Furthermore, the TME encompasses a biochemical facet rich in immune-related molecules, including immune checkpoints, cytokines, chemokines, and growth factors [38], [39]. Recent studies also identify nerves and neuro-associated factors, including neurotransmitters, neuropeptides, and nerve growth factors, as key TME components [40], [41]. Extracellular vesicles (EVs, such as exosomes) and their cargoes further mediate vital intercellular communication in the TME [40]. Overall, the TME is highly dynamic and heterogeneous, varying between tumor types and within different regions of the same tumor [42], [43].

The TME significantly influences the treatment response of RPT [44]. For example, hypoxia can reduce RPT efficacy, while good vascularization aids RPT distribution [45]. An immunosuppressive TME may hinder anti-tumor immunity, affecting RPT outcomes [46]. The TME not only influences RPT effectiveness but is also altered by treatment itself [47]. Moreover, microfluidic systems significantly enhance *in vitro* RPT testing by accurately simulating TME physical and biochemical traits [48], offering unique advantages in assessing targeted delivery, penetration efficiency, and drug resistance. Future advances will require integrating bioluminescence and radiometric detection to optimize their applications.

IT activates the patient’s immune system to target cancer cells, primarily through ICIs, chimeric antigen receptor (CAR)-T cell therapy, non-specific immune stimulation, oncolytic viruses, and vaccines [49], [50], [51], [52], [53]. RPT-IT combinations show synergistic effects for better tumor control. Studies indicate that this synergy largely depends on TME changes and interactions [15], [54]. RPT enhances tumor blood flow, triggers anti-tumor immunity, and disrupts immunosuppression, thereby boosting IT efficacy [55], [56], [57]. Additionally, RPT can alter the function of immunosuppressive cells within the TME, thereby alleviating immune suppression and enhancing the efficacy of IT [58], [59]. Conversely, IT can improve the efficacy of RPT. For instance, programmed cell death protein 1 (PD-1)/PD-L1 inhibitors can alleviate the immunosuppressive state of T cells, allowing more active T cells to infiltrate the tumor site [60], thereby enhancing the clearance of residual tumor cells following RPT-induced cell death. Additionally, CAR-T cell therapy can target specific tumor antigens, activating and expanding a substantial number of highly cytotoxic T cells. These T cells can further attack and eliminate any remaining cancer cells within the TME following RPT [61]. In summary, the TME may serve as a bridge between RPT and IT, facilitating their synergistic effects.

This review examines TME-RPT interaction mechanisms and preclinical/clinical advances in RPT-IT combinations over the past 15 years, using PubMed/Medline, Web of Science, and citation tracing. Therefore, this review aims to address the following key questions. (1) How does RPT remodel the TME to promote synergy with ITs? (2) Which TME components critically limit or enhance RPT efficacy? (3) How can TME-targeted combination strategies be designed to overcome current challenges? By synthesizing preclinical and clinical evidence, we summarize RPT-TME mechanisms and RPT-IT synergy, clarify the TME’s central role, highlight research gaps, and propose a framework for TME-guided combination therapy.

## Interaction between the tumor microenvironment and radiopharmaceutical therapy

**Fig. 1:**
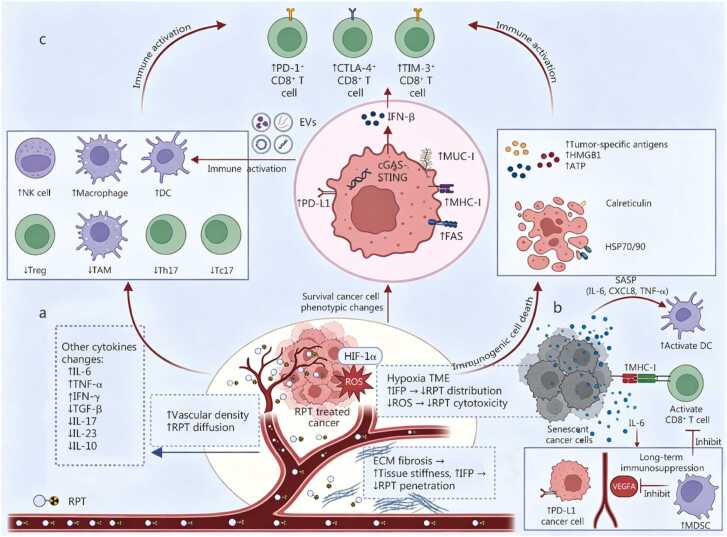
Direct and indirect interactions between radiopharmaceutical therapy (RPT) and components of the tumor microenvironment (TME). **a** TME impacts RPT delivery and cytotoxicity: increased vascular density may enhance RPT drug diffusion; however, hypoxic regions exhibit reduced RPT distribution due to aberrant vasculature and elevated IFP. Hypoxia also diminishes ROS generation, impairing RPT cytotoxicity. ECM fibrosis increases tissue stiffness and IFP, further hindering RPT penetration and convection. Other cytokines (e.g., IL-6, TNF- α, IFN-γ, TGF-β, IL-17, IL-23, IL-10) contribute to therapy resistance. **b** RPT-induced senescent cancer cells upregulate immunostimulatory receptors (MHC-I), enhancing immunogenicity and CD8⁺ T cell recognition. Senescent cells secrete a proinflammatory SASP (e.g., IL-6, CXCL8, TNF-α), which activates antigen-presenting DCs and CD8⁺ T cells. However, persistent SASP factors (e.g., IL-6) upregulate immune checkpoint PD-L1 on tumor cells, promoting immune evasion. Additionally, IL-6 recruits MDSCs, suppressing CD8⁺ T cell function, while SASP-derived angiogenic factors (e.g., VEGFA) are simultaneously inhibited. **c** RPT-treated cancer cells undergo ICD, releasing tumor-specific antigens, HMGB1, and ATP. Surviving cancer cells exhibit phenotypic changes, including upregulated PD-L1, MHC-I, MUC-1, and Fas expression, alongside IFN-β production via cGAS-STING pathway activation. RPT-induced EVs derived from cancer cells can carry double-stranded DNA, DNA damage response pathway-related gene transcripts, or immune checkpoint molecules, exerting anti-tumor effects by activating immune cells. Innate immune cell infiltration increases (e.g., NK cells, macrophages, DCs), while immunosuppressive populations (Tregs, TAMs, Th17, Tc17) decline. These changes collectively enhance immune activation, expanding cytotoxic CD8⁺ T cells (PD-1⁺/CTLA-4⁺/TIM-3⁺ subsets) and their tumor-killing capacity. IFP. Interstitial fluid pressure; ROS. Reactive oxygen species; ECM. Extracellular matrix; IL. Interleukin; TGF. Transforming growth factor; SASP. Senescence-associated secretory phenotype; CXCL. C-X-C motif chemokine ligand; DCs. Dendritic cells; PD-L1. Programmed cell death ligand 1; MDSCs. Myeloid-derived suppressor cells; MHC. Major histocompatibility complex; VEGFA. Vascular endothelial growth factor A; ICD. Immunogenic cell death; HMGB1. High-mobility group box 1; ATP. Adenosine triphosphate; Fas. Fas cell surface death receptor; cGAS. Cyclic GMP-AMP synthase; STING. Stimulator of interferon genes; IFN. Interferon; TNF. Tumor necrosis factor; EVs. Extracellular vesicles; DNA. Deoxyribonucleic acid; NK. Natural killer; Tregs. Regulatory T cells; TAM. Tumor-associated macrophage; Th. T helper cells; Tc. Cytotoxic T cells; CTLA. Cytotoxic T-lymphocyte-associated protein; TIM. T-cell immunoglobulin and mucin domain-containing protein; HSP. Heat shock protein; HIF. Hypoxia-inducible factor.

The efficacy of RPT is determined not solely by its direct cytotoxic effects on tumor cells but also by its dynamic interactions with the cellular and molecular components of the TME. Firstly, RPT can induce senescence of tumor cells within the TME. Furthermore, the interplay between RPT and various elements of the TME, including immune cells, cytokines, immune checkpoints, immune-related molecules, EVs, vasculature, hypoxia, and the barrier function of the ECM, constitutes a complex feedback network. This network is both regulated by the effects of RPT and, in turn, impacts the delivery efficiency and biological actions of RPT (Fig. 1).

### Cellular senescence and radiopharmaceutical therapy

Cellular senescence, a stress response mechanism, is distinguished by several characteristics, including cell cycle arrest, a distinctive morphological flattening, increased expression of cyclin-dependent kinase inhibitors such as p16 and p21, enhanced activity of senescence-associated β-galactosidase (SA-β-Gal), and the accumulation of the senescence-associated secretory phenotype (SASP) [62], [63], [64], [65]. The SASP consists of chemokines, growth factors, cytokines, matrix metalloproteinases, and other bioactive molecules [66]. SASP remodels the TME, enabling tumors to evade immune destruction [66], [67]. Some studies also suggest that SASP can hinder tumor progression by enhancing immune surveillance [67], [68], [69], and inducing tumor cell senescence has increasingly been recognized as a favorable therapeutic strategy for tumors [62], [70]. Next, we highlight several studies that reveal RPT can induce tumor cell senescence through multiple mechanisms, thereby exerting therapeutic effects [71], [72], [73], [74].

β-particle radiation generates reactive oxygen species (ROS) and induces DNA single-strand breaks, leading to sublethal cellular damage and a notable increase in SA-β-Gal activity between 9–14 d post-treatment, which triggers irreversible cellular senescence [71]. This process is characterized by reduced Ki-67 proliferation markers and tumor volume shrinkage, demonstrating that senescence is a key mechanism by which RPT suppresses tumor growth [71]. Targeted radioligand delivery via specific receptors significantly enhances the depth of DNA damage and the efficacy of senescence induction. ^177^Lu-satoreotide tetraxetan produces significant DNA double-strand breaks upon targeting the somatostatin receptor subtype 2 (SST2) and entering tumor cells, triggering irreversible cell cycle arrest and a senescent phenotype [72]. Specifically, this manifests as the loss of mitotic activity in cancer cells, cytoplasmic vacuolation, and the absence of proliferation marker Ki-67 and DNA damage marker phosphorylated histone H2AX (γH2AX) expression. Notably, 20% of tumors exhibited complete senescence after four cycles of ^177^Lu-satoreotide tetraxetan treatment. This senescent effect correlates with the drug’s high tumor uptake rate (3.5 MBq/g), cumulative radiation dose, and persistent DNA damage, ultimately inhibiting tumor recurrence and prolonging survival time (68 d vs. 43–48 d) [72].

Further investigation demonstrates that cellular senescence is not merely a response to RPT but the key mechanism underlying its durable therapeutic efficacy. A study found that the efficacy of ^177^Lu-DOTATATE radioligand therapy is closely linked to its induction of cellular senescence [73]. Specifically, successfully treated tumors demonstrated sustained accumulation of γH2AX signals relative to ^177^Lu retention during the critical time window (days 5–7), which was primarily driven by delayed induction of cellular senescence rather than apoptosis [73]. ^177^Lu-DOTATATE dose-dependently increased the proportion of SA-β-Gal-positive cells, with more than 80% of highly γH2AX-expressing cells exhibiting a senescent phenotype. *Ex vivo* tumor tissue analysis further revealed that SA-β-Gal-expressing senescent regions colocalized with prolonged ^177^Lu retention, persisting for up to 42 d post-treatment. The study concludes that cellular senescence is not only the molecular basis of ^177^Lu treatment response but also the key mechanism underlying its therapeutic efficacy and the optimal treatment outcome.

Another study synthesized a ^223^Ra/Ba single-atom nanozyme (SAzyme), where the integration of ²²³RaCl₂ with the SAzyme increased the local concentration of reactive species, thereby accelerating catalytic reactions and enhancing senescence induction [74]. The ^223^Ra/Ba SAzyme triggers irreversible DNA damage, initiating cellular senescence via the p53-p21 pathway. In the early stages, senescent cancer cells produce alarmins, activate interferon signaling, and upregulate major histocompatibility complex class I (MHC-I) expression, inducing robust anti-tumor immunity mediated by dendritic cells (DCs) and CD8⁺ T cells, which temporarily suppresses tumor growth. However, prolonged accumulation of SASP factors may promote immune evasion by recruiting myeloid-derived suppressor cells (MDSCs), which inhibit CD8⁺ T-cell activity [74]. To counteract the adverse effects of persistent senescent cells in tumors, a potential strategy involves eliminating them through PD-L1 immune checkpoint inhibition. PD-L1 inhibitors not only clear senescent cells but also block tumor cell immune evasion, thereby reducing the likelihood of tumor recurrence and metastasis [74].

α-particle-targeted therapy, such as ^211^At-AITM, pioneers a novel pathway for senescence-driven therapy by harnessing both superior DNA-damaging capability and oncogenic signaling modulation. It induces DNA double-strand breaks and persistently activates the DNA damage response, triggering upregulation of γH2AX expression, leading to cell cycle arrest in G2/M phase and permanent senescence (SA-β-Gal-positive) [75]. Concurrently, this treatment significantly downregulates expression of the oncoprotein metabotropic glutamate receptor 1 (*mGluR1*) (both mRNA and protein levels), inhibiting pro-tumor signaling pathways including mitogen-activated protein kinase/phosphoinositide 3-kinase/mammalian target of rapamycin (MAPK/PI3K/mTOR). Beyond direct cellular effects, the SASP is reprogrammed into an “immune-activated” state, characterized by increased secretion of proinflammatory factors [interleukin-6 (IL-6), C-X-C motif chemokine ligand 8 (CXCL8), tumor necrosis factor-α (TNF-α)] to enhance anti-tumor immunity, while pro-angiogenic factor and pro-metastatic factor remain persistently suppressed. Collectively, this reprogrammed SASP achieves durable therapeutic efficacy against mGluR1-positive tumors [75].

### Immune cell, cytokine, and radiopharmaceutical therapy

β-emitters (such as ^90^Y, ^177^Lu, ^131^I) leverage their relatively long radiation range to elicit a series of common immunomodulatory effects. These primarily include the depletion of immunosuppressive components such as reducing regulatory T cells (Tregs) and tumor-associated macrophages (TAMs) [58], [59], and the recruitment of cytotoxic immune effector cells, particularly enhancing the infiltration and function of CD8⁺ T cells and natural killer (NK) cells through the activation of the cyclic GMP-AMP synthase/stimulator of interferon genes (cGAS/STING) pathway [59], [76], [77]. Accompanying these cellular changes, a key remodeling of the cytokine network occurs. On one hand, treatment promotes the production of proinflammatory and immune-activating factors, such as the upregulation of interferon-β (IFN-β) by ^90^Y-NM600 [76] and increased serum levels of TNF-α and IFN-γ with ^131^I combined with CpG [78]. On the other hand, it significantly reduces various inhibitory cytokines, as observed in differentiated thyroid cancer patients, where levels of IL-17, IL-23, IL-10, and transforming growth factor-β1 (TGF-β1) markedly decreased after ^131^I therapy [79]. Additionally, ^131^I combined with immune adjuvants can induce the generation of effector memory T cells, establishing long-term immunological memory [78]. In contrast, α emitters (such as ^123^RaCl₂), due to their high linear energy transfer (LET) and short-range physical characteristics, exhibit distinct immunomodulatory features. The dense ionization damage they induce can trigger strong immune signals, exemplified by elevated plasma IL-6 levels post-treatment [80]. However, α-particles may also foster an immunosuppressive milieu, as evidenced by an increase in the proportion of Tregs and MDSCs during therapy [81], suggesting that the cytokine environment they evoke may be more complex.

### Immune checkpoint, immune-related molecule, and radiopharmaceutical therapy

RPT regulates immune checkpoints and related molecules, which is one of its core mechanisms for remodeling the TME [81], [82], [83], [84]. By inducing immunogenic cell death (ICD) and inflammatory signals, RPT commonly upregulates the expression of immune checkpoint molecules [85], [86]. This commonality is confirmed both by β-emitters (such as ^90^Y-NM600) upregulating PD-1, cytotoxic T-lymphocyte-associated protein 4 (CTLA-4), and lymphocyte activation gene-3 on CD8⁺ T cells and PD-L1 on myeloid cells in models of prostate cancer, melanoma, etc. [82], [83], [84], and by α-emitters (such as ^223^Ra) increasing T cells expressing T-cell immunoglobulin and mucin domain-containing protein 3 (TIM-3), PD-L1, and PD-1 during treatment [81]. The key upstream pathway involves radiation-induced IFN-γ release and subsequent Janus kinase/signal transducer and activator of transcription (JAK/STAT) signaling activation. For example, ^177^Lu-LNC1004 and ^225^Ac-9079 upregulate PD-L1 on tumor or immune cells, respectively, through the JAK/STAT pathway [85], [86].

^131^I-CF01012 exerts pleiotropic immunomodulation by inducing calreticulin translocation, damage-associated molecular patterns (DAMPs), release of high-mobility group box 1 (HMGB1)/adenosine triphosphate (ATP), and type I IFN secretion [87], which enhances RPT efficacy via CD8⁺ T cell recruitment, activation, and cytotoxicity [88]. *In vitro* radiation with radium-223 enhances CD8^+^ T cell-mediated killing of prostate, breast, and lung cancer cells through mucin-1 (MUC-1), brachyury, and carcinoembryonic antigens (CEAs), accompanied by upregulated expression of MHC-I and calreticulin on tumor cell surfaces [89]. Chakraborty *et al*. [90] reported that ^153^Sm-ethylenediamine tetramethylene phosphonate (EDTMP) upregulated at least two of the following immune-related molecules: Fas cell surface death receptor/CEA/MUC-1/MHC-I/intercellular adhesion molecule 1 (ICAM-1), in each of 10 tumor cell lines examined; in addition, in LNCaP cells, this treatment enhanced cytotoxic T lymphocyte (CTL)-mediated killing. The mesothelin (MSLN)-targeted thorium-227 conjugate, BAY 2287411, has been shown to upregulate DAMPs, including calreticulin, heat shock protein (HSP)70, HSP90, and HMGB1, in OVCAR-3 cells *in vitro* [91]. In CEA-transgenic mice transplanted with MC38-CEA^+^ tumor cells, Yttrium-90-labeled COL-1 antibody treatment significantly upregulated cell-surface Fas expression compared to non-irradiated tumor cells [90]. Treatment with 1.5 mCi of ^188^Re-6D2 (which binds to melanin) resulted in tumor growth inhibition in the A2058 human melanoma xenograft model, along with a significant increase in complement C3 [92].

Direct comparative studies indicate that α-particle therapy (^225^Ac) induces more robust upregulation of PD-1 expression on CD8⁺ T cells and PD-L1 expression on MDSCs compared to β-particle therapy (^177^Lu) [93]. A recent study employing magnetic resonance imaging-based 3D spatiotemporal models has elucidated the differential impacts of α therapy [^225^Ac-prostate-specific membrane antigen (PSMA)] and β therapy (^177^Lu-PSMA) on the TME in prostate cancer [94]. α-particles, characterized by high LET, induce dense DNA double-strand breaks and robust ICD, evidenced by calreticulin exposure and HMGB1 release. These particles achieve a 4.2-fold higher accumulation in PSMA-targeted regions *in vitro* compared to β therapy. In contrast, β-particles predominantly cause sublethal damage and activate pro-survival pathways, suggesting that while α therapy is preferable for smaller tumors requiring intense ICD effects, β therapy may be more effective for managing TME heterogeneity in larger tumors [94].

### Vasculature, hypoxia, and radiopharmaceutical therapy

The permeability of vascular endothelial cells, which exhibit varying structural subtypes, significantly influences the transport and biodistribution of RPTs. For instance, a continuous endothelium, characteristic of tissues such as skeletal muscle, myocardium, skin, and bone, restricts the transport of macromolecules. Conversely, fenestrated or discontinuous endothelium, often found in other tissues, facilitates the biodistribution of RPTs [95], [96]. In addition to endothelial structural types, the density and spatial distribution of blood vessels critically affect the local distribution and accumulation of drugs in tumors, with an increased vascular density typically enhancing tracer diffusion into tumor tissues [97]. To quantitatively assess this effect, this investigation utilized a spatiotemporal multiscale mathematical model to study the delivery of ¹⁸F-fluoromisonidazole (FMISO) in vascularized solid tumors, particularly focusing on the impact of varying microvascular densities (MVDs) on tracer transport. The findings indicated that tumors with a uniform vascular distribution, attributed to a higher MVD, exhibited enhanced release and diffusion of ¹⁸F-FMISO, resulting in significantly higher cellular uptake rates and standardized uptake value metrics compared to tumors with a heterogeneous vascular distribution where MVD was lower [97]. Another mathematical model further demonstrates that tumor MVD and quality jointly regulate the spatiotemporal distribution of RPTs, where higher capillary permeability significantly enhances drug accumulation and therapeutic efficacy [98]. These mechanisms are not only critical in treatment but are also reflected in diagnostic applications. For instance, the RPT ^99m^Tc-3PRGD2 achieves highly specific imaging of lesions in lung cancer models by targeting αvβ3 integrin, a biomarker of neovascularization, with uptake intensity showing a strong positive correlation (*r*=0.88) with tumor microvessel density [99]. However, it is noteworthy that increased vascular permeability does not always confer benefits. A study explored the multiscale transport of ^177^Lu-PSMA-617 in prostate tumors using a similar spatiotemporal mathematical model. The results indicated that characteristics of tumor microvasculature, such as hyperpermeability and the absence of lymphatic drainage, enhanced drug extravasation but also led to interstitial fluid accumulation, significantly raising interstitial fluid pressure (IFP) in the tumor core. This elevated IFP inhibited drug penetration to central tumor regions, resulting in suboptimal core dosage [100].

Hypoxia, a state of inadequate oxygen supply, profoundly affects the biodistribution and pharmacokinetics of RPTs across different tumor types. It is a common feature of solid tumors, contributing to treatment resistance and poor clinical outcomes [101], [102]. The biodistribution of RPTs is influenced by oxygenation status; for instance, hypoxic regions receive significantly lower radiation doses in PSMA-targeted radioligand therapy for prostate cancer [103]. Hypoxia further indirectly impairs drug delivery through physical mechanisms. Using convection-diffusion-reaction modeling, Birindelli *et al*. [100] demonstrated that chronic hypoxia disrupts transport efficiency by altering vascular distribution, including the presence of “leaky” vasculature, and by modifying IFP in necrotic tumor areas. Remarkably, certain hypoxia-avid tracers can exploit this condition for diagnostic purposes. ⁹⁹ᵐTc-labeled HL91 (a nitroimidazole-based hypoxia imaging agent) undergoes selective nitroreduction by intracellular reductases under hypoxia, leading to its selective accumulation in hypoxic regions, which manifests as localized signal enhancement on positron emission tomography (PET) or single photon emission computed tomography (SPECT) imaging [104].

Beyond affecting transport, hypoxia directly undermines therapeutic efficacy. *In vitro* radiobiological study confirms that β-emitters exhibit oxygen-dependent cytotoxicity, relying on ROS-mediated indirect DNA damage [46]. Hypoxia reduces ROS generation, weakening cytotoxic effects and diminishing treatment response. Hypoxia also increases the chance of cells repairing DNA, as oxygen is required to make the DNA damage permanent. In contrast, α-particles and Auger electrons induce direct DNA strand breaks independent of oxygen, maintaining potent killing efficiency even in hypoxic conditions [46]. These seemingly contradictory observations underscore the need to match RPTs to the TME, such as using high-LET radionuclides against hypoxic areas, when optimizing therapies. Notably, hypoxia not only reduces lethal DNA damage from RPTs but also upregulates protective signaling and survival factors, enhancing tumor cell radioresistance [105].

### Extracellular matrix and radiopharmaceutical therapy

The ECM, composed of macromolecules such as collagen, elastin, proteoglycans, and glycoproteins, not only provides structural support but also regulates key cellular functions, including adhesion, migration, proliferation, and survival [106]. In solid tumors, the ECM undergoes pathological remodeling, forming a dense physical barrier that impedes the uniform distribution of RPTs, reduces therapeutic efficacy, and contributes to drug resistance [107], [108].

More importantly, the composition and structural properties of the ECM critically affect tissue penetration and retention of RPTs [109]. A study reveals that ECM-mediated drug distribution varies substantially depending on molecular formulations, as reflected in the distinct tissue distribution observed between free ¹⁷⁷Lu ions and conjugates like methylenediphosphonate or monoclonal antibodies [110]. Optimizing drug design can enhance targeting efficiency by tuning interactions with the ECM and cell membranes [111]. To gain a deeper understanding of the mechanisms underlying this physical barrier, a study proposed a multi-scale computational model based on real tumor images, revealing that high-stiffness ECM hinders drug convective transport by reducing interstitial hydraulic conductivity and increasing IFP, resulting in inadequate drug penetration into deep tumor regions [112].

The ECM barrier effect primarily manifests as increased tissue stiffness and IFP, thereby hindering the diffusion and convective transport of RPT. Meanwhile, elevated IFP drives drugs outward through convection rather than penetration into the tumor core, representing a key physical barrier that reduces therapeutic efficacy [100]. Collagen deposition and fibrosis stiffen the ECM, impeding interstitial flow and elevating tissue interstitial fluid pressure (TIFP). High TIFP, in turn, collapses vessels, suppresses ECM remodeling, and perpetuates a RPT drug-delivery-limiting feedback loop [113]. Notably, RPT itself also exerts effects on the ECM. For instance, RPT-induced damage to ECM proteins can profoundly affect tissue structure, composition, and function, which in turn impacts the effectiveness of RPT [114].

### Extracellular vesicles and radiopharmaceutical therapy

EVs in TME are lipid bilayer-enclosed particles released by cells that contain cytoplasmic contents. They can transport cargoes (DNA, RNA, miRNA, proteins, lipids) from donor cells to recipient cells, thereby mediating intercellular communication and potentially regulating their physiological functions [115]. EVs can exert anti-tumor effects by activating immune cells [116], but may also carry pro-tumor components [117]. Tumor-derived EVs primarily carry double-stranded DNA, which may be involved in immune regulation [118]. A study has found that EVs induced by the ^125^I-TA99 monoclonal antibody exhibit significant cytotoxicity [119]. Vardaki *et al*. [120] analyzed EVs in the blood of metastatic castration-resistant prostate cancer (mCRPC) patients treated with ^223^Ra and discovered that these EVs were enriched with DNA damage response pathway-related gene transcripts and immune checkpoints.

**Fig. 2:**
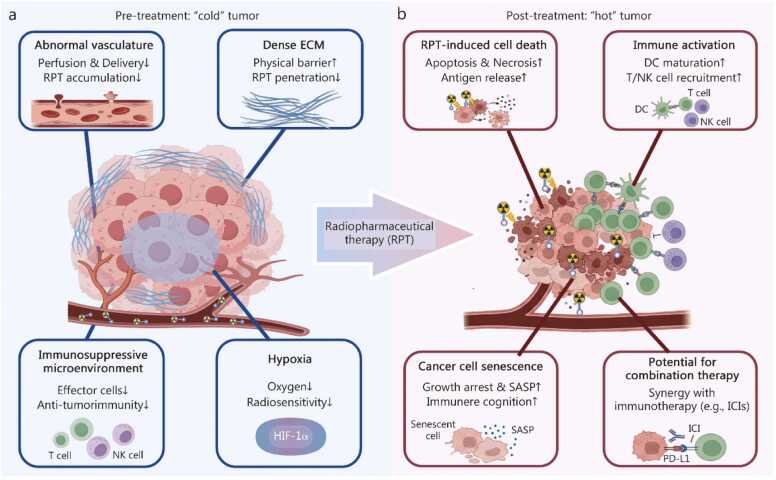
Changes in tumor and tumor microenvironment components before and after radiopharmaceutical therapy (RPT). **a** The constraints imposed by the pre-treatment “cold” tumor on RPT. **b** Following RPT treatment, tumor volume is reduced, the tumor converts to a “hot” tumor, and there is potential for synergistic effects with immunotherapy. ↑: Activation/Promotion/lncrease; ↓: Inhibition/Decrease; ECM. Extracellular matrix; SASP. Senescence-associated secretory phenotype; HIF-1α. Hypoxia-inducible factor 1α; ICI. Immune checkpoint inhibitor; PD-L1. Programmed death-ligand 1; NK cell. Natural killer cell; DC. Dendritic cell.

In conclusion, RPT markedly induces tumor cell senescence, primarily through mechanisms of cell cycle arrest and the formation of SASPs. SASPs play a dual regulatory role within the TME. The targeted reprogramming of SASPs to an “immune-activated” state constitutes a novel approach to augmenting the efficacy of RPT, providing a critical theoretical basis for the development of combination therapies. This section also reviews the bidirectional interactions between RPT and the TME. On one hand, RPT reprograms the TME into an immunostimulatory state by altering the balance of immune cell subsets and releasing immunogenic signals. On the other hand, intrinsic characteristics of the TME, such as hypoxia and ECM densification, significantly limit the biodistribution, cytotoxicity, and therapeutic response of RPT (Fig. 2**;** Table 1). This dynamic interplay offers new insights for optimizing precision dosing strategies and enhancing the synergistic potential of RPT in combination with IT.

**Table 1:** Effects of different tumor microenvironment components on radiopharmaceutical therapy (RPT) and corresponding mechanisms.

**TME components**	**Positive effects on RPT**	**Negative effects on RPT**
Vasculature	Promotes delivery: a dense and uniformly distributed vascular network enhances RPTs’ perfusion; Provides a therapeutic window: fenestrated or discontinuous endothelium promotes the biodistribution of RPTs	Limits penetration: high vascular permeability, coupled with a lack of lymphatic drainage, leads to abnormally elevated IFP, which hinders RPTs’ penetration into the tumor core; Uneven distribution: drugs tend to accumulate around blood vessels, leading to insufficient dosing in the tumor core
Hypoxia	Provides diagnostic targets: hypoxia-specific tracers (e.g., ^99m^Tc-HL91) can be utilized for imaging diagnosis	Weakens efficacy: the cytotoxic effect of β-particles largely relies on radiation-generated reactive oxygen species (ROS). A hypoxic environment reduces ROS production, significantly diminishing this effect; Induces radioresistance: hypoxia activates signaling pathways such as HIF-1α, upregulating genes involved in DNA repair and cell survival, thereby enhancing the intrinsic resistance of tumor cells; Limits delivery: chronic hypoxia alters vascular distribution and modifies IFP in necrotic tumor areas, thereby hindering drug delivery
Extracellular matrix (ECM)	-	Constitutes a physical barrier: a dense and stiff ECM severely impedes the passive diffusion of drug molecules; Limits convective transport: ECM thickening reduces tissue hydraulic conductivity. Combined with high IFP, this suppresses convective drug transport, resulting in insufficient penetration depth
Immunosuppressive microenvironment	-	Limits delivery: factors secreted by immunosuppressive cells (e.g., TGF-β, IL-10) can promote vascular abnormalities and ECM fibrosis, thereby physically limiting drug delivery; Weakens efficacy: weakens the anti-tumor immune response induced by RPT. Therapeutic efficacy is thus largely confined to direct radiation damage, and it creates unfavorable conditions for combination with immunotherapy

TME. Tumor microenvironment; IFP. Interstitial fluid pressure; HIF-1α. Hypoxia-inducible factor 1α; DNA. Deoxyribonucleic acid; TGF-β. Transforming growth factor-β; IL-10. Interleukin-10

## The role of tumor microenvironment in the combination of radiopharmaceutical therapy and immunotherapy

IT is one of the most important strategies in cancer treatment. It primarily works by reversing the immune suppression of tumor cells, precisely recognizing tumor cells, activating immune cells, and improving the TME to enhance immune attacks against tumors [121]. Cancer IT mainly includes ICIs, CAR-T cell therapy, and cancer vaccines [122]. However, some tumors can evade immune attacks through complex mechanisms, thereby reducing the efficacy of IT and leading to resistance or even treatment failure in patients [123]. Studies have shown that the combination of various ITs with RPTs can improve immune responses, enhance therapeutic efficacy, overcome resistance, and increase patient survival rates in multiple cancer types [15], [54]. In this section, we will explore how the various components of the TME contribute to the synergistic effects of combining RPTs and ITs in cancer treatment.

### Radiopharmaceutical therapy and immune checkpoint inhibitors

**Fig. 3:**
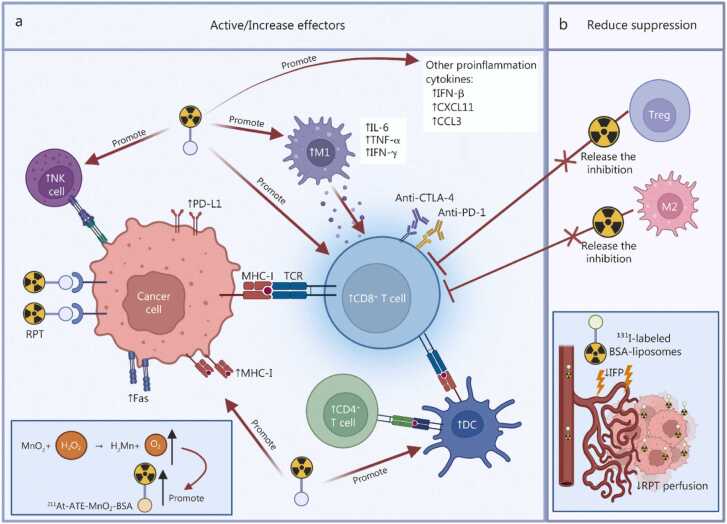
Synergistic effects of combined radiopharmaceutical therapy (RPT) and immune checkpoint inhibitors in the tumor microenvironment. **a** Enhanced anti-tumor immunity: combination therapy potently augments tumor-specific immune responses. RPT directly targets tumor cells, upregulating surface expression of MHC-I, Fas, and PD-L1, thereby promoting CD8⁺ T cell activation through T cell receptor (TCR). Increased infiltration of innate immune cells, including DCs, NK cells, and M1-polarized macrophages, further amplifies immune surveillance. DCs enhance cross-presentation of tumor antigens to CD8⁺ T cells, while M1 macrophages secrete proinflammatory cytokines (IL-6, TNF-α, IFN-γ) to bolster T cell priming. Elevated levels of other immunostimulatory mediators (IFN-β, CXCL11, CCL3) contribute to a proinflammatory TME. Concurrently, ICIs (anti-PD-1/anti-CTLA-4) block inhibitory receptor-ligand interactions on CD8⁺ T cells, enhancing their cytotoxic potential. Additionally, MnO₂ in ²¹¹At-ATE-MnO₂-BSA nanoparticles catalytically decomposes H₂O₂, alleviating hypoxia and radiosensitizing tumors. **b** Attenuation of immunosuppression: RPT counteracts TME-driven immune evasion by depleting immunosuppressive Tregs and M2-polarized macrophages, thus restoring CD8⁺ effector T cell function. Moreover, ¹³¹I-labeled BSA-liposomes disrupt abnormal tumor vasculature, reducing IFP and improving RPT perfusion. MHC-I. Major histocompatibility complex class I; PD-L1. Programmed death ligand 1; DCs. Dendritic cells; NK cells. Natural killer cells; IL-6. Interleukin-6; TNF-α. Tumor necrosis factor-α; IFN-γ. Interferon-γ; IFN-β. Interferon-β; CXCL11. C-X-C motif chemokine ligand 11; CCL3. C-C motif chemokine ligand 3; PD-1. Programmed cell death protein 1; CTLA-4. Cytotoxic T-lymphocyte-associated protein 4; MnO₂. Manganese dioxide; H₂O₂. Hydrogen peroxide; Tregs. Regulatory T cells; IFP. Interstitial fluid pressure; BSA. Bovine serum albumin; M1. M1-polarized macrophages; M2. M2-polarized macrophages.

ICIs enhance the immune system’s attack on cancer cells by blocking the binding of immune cell surface receptors to their corresponding ligands, thereby reversing immune suppression and precisely recognizing and attacking tumor cells [124], [125]. Preclinical and clinical evidence suggest that the combination of RPTs with ICIs can exert synergistic effects by modulating the TME, both enhancing anti-tumor immunity and alleviating immune suppression (Fig. 3), thereby improving tumor responses, reducing metastasis, and enhancing therapeutic efficacy [85], [126].

The primary role of RPTs is to initiate and amplify anti-tumor immune responses by inducing ICD in tumor cells, releasing tumor antigens and DAMPs, thereby activating antigen-presenting cells and initiating T-cell immunity. Examples of this mechanism are demonstrated in models combining ^131^I-labeled ICF01012 and ^90^Y-labeled granzyme B with ICIs [56], [87]. More importantly, radiation can directly activate intracellular signaling pathways. For instance, ^90^Y-NM600 and [^211^At]MM4 can activate the cGAS/STING pathway, promoting the production of type I IFN and proinflammatory chemokines, which efficiently recruit effector cells such as CD8⁺ T cells and NK cells to infiltrate the tumor [76], [127]. This immune-activating effect has been validated with various agents. For example, both ^211^At-ATE-MnO₂-BSA and ^177^Lu-EB-RGD significantly increase intratumoral CD8⁺ T cell infiltration and the secretion of effector factors like IFN-γ [128], [129].

However, immune activation alone is often insufficient to overcome tumor immune evasion because radiation, while activating immunity, may also trigger immunosuppressive mechanisms. For instance, ^131^I-labeled ICF01012 treatment increases the number of Tregs and CTLA-4 expression while activating CD8⁺ T cells and NK cells [87]. Similarly, after enhancing CD8⁺ T cell infiltration, ^225^Ac-labeled CS1-targeted antibodies also upregulate PD-L1 expression on tumors [130]. The ICIs can precisely remove these inhibitory signals and block the immune feedback mechanisms triggered by the radiopharmaceutical or the tumor itself. The realization of synergistic effects highly depends on the sequential and complementary application of the two therapies. After radiopharmaceuticals transform “cold” tumors into inflammatory “hot” tumors, ICIs can prevent the exhaustion of newly recruited T cells and reverse pre-existing immunosuppression. For example, combining anti-CTLA-4 antibodies with ^131^I-labeled ICF01012 treatment effectively blocks the Tregs and CTLA-4-mediated tolerance signals induced by the therapy, significantly prolonging survival and downregulating T-cell exhaustion markers [87]. In melanoma, combining ^177^Lu-DOTA-PEG4-LLP2A with ICIs (blocking PD-1/PD-L1 or CTLA-4) alleviates immunosuppression and improves survival/apoptosis in mice [131]. Similarly, ^225^Ac-PSMA617 induces ICD and activates T cells; combining it with PD-1 inhibitors can reverse PD-L1-mediated T-cell exhaustion, significantly prolonging time to progression and survival in a prostate cancer model [132]. Direct modulation of immunosuppressive immune cell populations is also effective; for instance, ^90^Y-NM600 can directly suppress Tregs while activating the STING pathway to increase CD8⁺ T cell infiltration [76]. Furthermore, improving the physicochemical properties of the TME can indirectly overcome immunosuppression. For example, ^211^At-ATE-MnO₂-BSA improves tumor hypoxia and the metabolic inhibitory environment, synergizing with anti-PD-L1 therapy to suppress both primary and distant tumors [128].

In recent years, strategies utilizing novel material delivery platforms to combine RPT and IT have increasingly become a research hotspot in oncology. The key advantage of this approach lies in its ability to achieve synergistic effects across multiple therapeutic mechanisms through advanced material design, thereby overcoming the limitations of single-modality treatments [133], [134], [135]. Therefore, we have also summarized how different components of the TME contribute to this synergistic interplay. Spatial immunomodulation mediated by novel materials forms the foundation for achieving high-efficiency synergy. Through sophisticated design, nanocarriers can optimize the enrichment and distribution of drugs at the tumor site and actively regulate the physicochemical properties of the TME. For instance, ^131^I-BSA-liposomes utilize radiation to damage tumor blood vessels, significantly enhancing vascular permeability. This not only allows the nanocarrier itself to penetrate deeper but also increases the accumulation of co-administered anti-PD-L1 antibodies in the tumor by 8-fold [136]. Meanwhile, smart materials like metal-organic frameworks co-delivering MSA-2^+^ can transport both radiosensitizing components (Hf₆) and immune agonists (the STING agonist MSA-2) to the same lesion, ensuring the simultaneous occurrence of RT and immune activation in space [137].

Under the precise regulation of these material platforms, the mechanisms of enhancing immune activation and relieving immunosuppression can be efficiently and synchronously executed. Material intervention often makes immune activation stronger and more durable. For example, the combination of a ferroptosis inducer with ^131^I synergistically amplifies ferroptosis and radiocytotoxicity through bidirectional regulation of glutathione peroxidase 4/solute carrier family 7 member 11, jointly inducing potent ICD and providing abundant antigens and danger signals for activating anti-tumor immunity [138]. Additionally, the presence of novel material platforms makes this “relief of suppression” more precise and effective. RPT itself (e.g., via ^131^I-BSA-liposomes) can upregulate PD-L1 expression on tumor cells, which increases tumor sensitivity to subsequent PD-1/PD-L1 blockade [136]. The inflammatory microenvironment created by material platforms, while recruiting effector T cells, may also recruit or retain immunosuppressive cells (e.g., MDSCs) [139]. Combining ICIs can specifically reverse this suppression [139], [140]. After treatment with radioactive gold NPs, anti-PD-1 antibodies effectively relieve the checkpoint inhibition on activated cytotoxic T cells, working together with the material RT to induce long-term immune memory [139], [140].

### Radiopharmaceutical therapy and CAR-T cell therapy

**Fig. 4:**
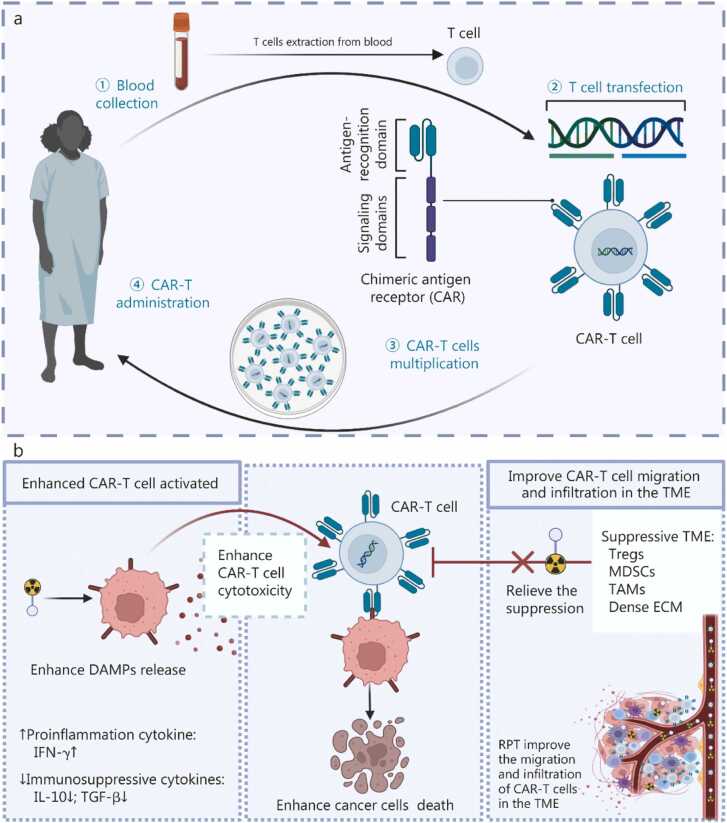
Immunological synergy between radiopharmaceutical therapy (RPT) and chimeric antigen receptor T cell (CAR-T) therapy in the tumor microenvironment. **a** Production of engineered CAR-T cells. **b** The combination of RPT and CAR-T cell therapy enhances anti-tumor efficacy through multiple mechanisms: CAR-T cells specifically recognize and eliminate tumor cells through their engineered antigen receptors. RPT promotes the release of DAMPs from tumor cells, enhancing antigen presentation and CAR-T cell activation. The combination increases proinflammatory cytokine secretion (e.g., IFN-γ) while reducing immunosuppressive factors (IL-10, TGF-β), creating a more favorable environment for CAR-T cell function. It targets immunosuppressive components (Tregs, MDSCs, TAMs, and dense ECM), alleviating their inhibitory effects on CAR-T cells. It improves CAR-T cell migration and infiltration into the tumor site by reducing physical and biochemical barriers. TME. Tumor microenvironment; DAMPs. Damage-associated molecular patterns; IFN-γ. Interferon-γ; IL-10. Interleukin-10; TGF-β. Transforming growth factor-β; Tregs. Regulatory T cells; MDSCs. Myeloid-derived suppressor cells; TAMs. Tumor-associated macrophages; ECM. Extracellular matrix.

CAR-T cell therapy is a form of adoptive cell IT that involves genetically modifying a patient’s T cells to specifically recognize and eliminate cancer cells. The key mechanism relies on engineering T cells to express a synthetic CAR, which fuses an extracellular antigen-binding domain (typically derived from monoclonal antibodies) with intracellular signaling domains, thereby activating T-cell cytotoxicity [141], [142]. After reinfusion, these modified CAR-T cells can target tumor-specific antigens and exert potent cell-killing effects while establishing long-term immune surveillance. Currently approved CAR-T therapies primarily target hematologic malignancies, but their efficacy in solid tumors remains limited due to immune suppression and T-cell exhaustion [61]. Emerging studies have shown that the TME can serve as a bridge to enhance the synergistic effects of combined RPT and CAR-T therapy, both by enhancing CAR-T cell activity and improving CAR-T cell trafficking and infiltration, thereby enhancing anti-tumor efficacy (Fig. 4) [143], [144], [145].

The TME enhances CAR-T cell efficacy via low-dose RPT. ^177^Lu and ^225^Ac radiation activate APCs via DAMPs, promote antigen exposure, and reduce Tregs, creating a favorable milieu for CAR-T cells. ^177^Lu and ^225^Ac radiation also boost CAR-T cytotoxicity via natural killer group 2 member D (NKG2D) pathway activation [143]. ^177^Lu-DOTATATE synergizes with CAR-T therapy by priming the TME, including IFN-γ secretion, death receptor upregulation (Fas/TNF-related apoptosis-inducing ligand receptor), M1 macrophage recruitment, and vascular normalization, enhancing tumor killing and CAR-T cell survival in hypoxia [144]. In small‑cell lung cancer, ^177^Lu weakens the immune barrier from MDSCs/TAMs by reducing IL-10/TGF-β, alleviating delta-like canonical notch ligand 3 (DLL3)-CAR-T suppression. It enhances matrix metalloproteinase-2/-9 activity to degrade collagen I/III and hyaluronic acid (HA), breaking the ECM barrier to boost CAR-T cell infiltration and expansion. After RPT, residual tumor cells continue to express high levels of DLL3, sustaining CAR-T targeting for further lesion clearance [145].

### Radiopharmaceutical therapy and cancer vaccines

**Fig. 5:**
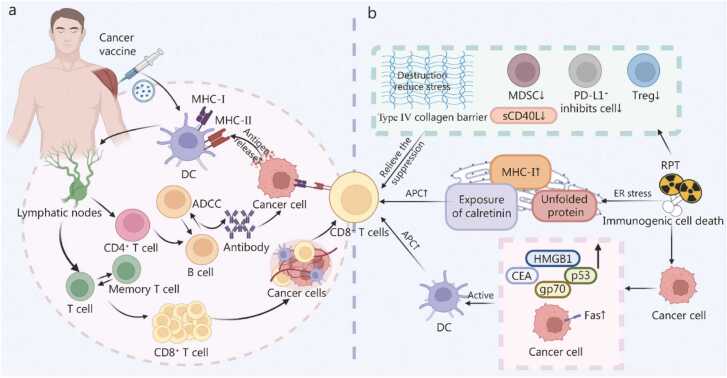
Synergistic mechanisms between radiopharmaceutical therapy (RPT) and cancer vaccines in modulating the tumor microenvironment. **a** Cancer vaccine-induced anti-tumor immunity cycle: upon administration, the cancer vaccine is taken up by DCs, which process and present tumor antigens via MHC class II (MHC-II) or cross-present through MHC class I (MHC-I). Antigen-loaded DCs migrate to lymph nodes to activate immune cells. Follicular DCs promote the generation of memory B cells and plasma cells, while activated B cells mediate tumor cell apoptosis through ADCC. Activated T cells proliferate and differentiate into memory T cells and cytotoxic CD8⁺ T cells, which infiltrate the TME to directly eliminate tumor cells or induce apoptosis. Immunogenic tumor cell death releases tumor-associated antigens and danger signals (e.g., DAMPs), amplifying the breadth and depth of subsequent immune responses. **b** RPT-enhanced anti-tumor immunity: RPT augments vaccine efficacy by inducing radiation-mediated ICD, releasing tumor antigens (CEA /gp70/p53) and damage-associated molecular patterns (e.g., HMGB1). It also upregulates Fas expression on cancer cells, enhancing DC activation and APC, thereby bolstering CD8⁺ T cell priming. Sublethal radiation triggers ER stress, calreticulin exposure, and MHC class I upregulation, improving CD8⁺ T cell recognition and antigen presentation efficiency. The ER stress-induced UPR further enhances immunogenicity, resulting in stronger tumor-killing capacity of vaccine-primed CD8⁺ T cells within the irradiated TME. Simultaneously, RPT alleviates TME immunosuppression by reducing MDSCs and PD-L1⁺ immunosuppressive populations; decreasing inhibitory factors such as sCD40L; counteracting Treg-mediated immune suppression; disrupting type IV collagen barriers and lowering interstitial pressure, thereby facilitating T cell infiltration. DCs. Dendritic cells; MHC. Major histocompatibility complex; ADCC. Antibody-dependent cellular cytotoxicity; TME. Tumor microenvironment; DAMPs. Damage-associated molecular patterns; ICD. Immunogenic cell death; APC. Antigen-presenting cell; ER. Endoplasmic reticulum; UPR. Unfolded protein response; MDSCs. Myeloid-derived suppressor cells; sCD40L. Soluble CD40 ligand; Tregs. Regulatory T cells; PD-L1. Programmed cell death ligand 1; HMGB1. High-mobility group box 1; CEA. Carcinoembryonic antigen; gp70. Glycoprotein 70; p53. Tumor protein p53; Fas. Fas cell surface death receptor.

Cancer vaccines are designed to stimulate the immune system to recognize and combat cancer cells. These vaccines operate by introducing tumor-associated antigens, neoantigens, or immune-stimulating components that activate T cells and other immune cells, thereby targeting malignant tumors [146]. Ongoing research focuses on the potential synergistic effects of combining cancer vaccines with ICIs, CAR-T therapy, or RT, including RPT, to enhance therapeutic outcomes. Presently, only a limited number of cancer vaccines, such as Sipuleucel-T for prostate cancer, have gained clinical approval. This review discusses the synergistic effects of TME components when cancer vaccines and RPT are administered concomitantly [147] (Fig. 5).

In triple-negative breast cancer, *RAS*-mutant lung cancer, and bone-metastatic models, ^223^Ra triggers endoplasmic reticulum stress via sublethal radiation, inducing immunogenic changes like calreticulin exposure and MHC-I upregulation. These enhance CTL recognition and antigen presentation, boosting TME immune sensitivity. Moreover, endoplasmic reticulum stress-driven unfolded protein response amplifies immunogenicity, which in turn improves vaccine-induced CTL tumor killing in radiation-exposed TME [89]. Similarly, in the CEA^+^ MC38 colon cancer model, the dynamic regulation of the TME mediates the synergistic therapeutic effects of RPT combined with a CEA-targeted vaccine. Specifically, RPT radiation upregulates Fas expression in tumor cells, thereby enhancing their susceptibility to vaccine-activated Fas ligand (FasL)-expressing CD8^+^ T cells, while also promoting the release of tumor antigens, including CEA, p53, and glycoprotein 70 (gp70). This remodeling significantly reduces T cell apoptosis (15% to 4%) and increases antigen-specific T cell infiltration by 3.2-fold, while simultaneously upregulating ICAM-1, disrupting the type IV collagen barrier, and reducing interstitial pressure, which collectively enhances antibody and T cell infiltration. Ultimately, 20% of mice achieved complete remission [90]. In the prostate cancer model, the components of the TME play a crucial regulatory role in the synergistic effects of ^153^Sm-EDTMP combined with the cancer vaccine [prostate-specific antigen (PSA)-TRICOM]. RPT induces ICD through radiation, releasing tumor antigens and damage-associated molecules (e.g., HMGB1), which activate DCs and enhance antigen presentation, laying the foundation for the vaccine to stimulate systemic T-cell responses. Furthermore, RPT reshapes the immunosuppressive nature of TME, reducing the proportion of MDSCs and PD-L1^+^ inhibitory cells, lowering the levels of the immunosuppressive factor soluble CD40 ligand (sCD40L), and alleviating Treg-mediated immune suppression. This, in turn, enhances the infiltration and tumor-killing capacity of vaccine-induced PSA-specific CD8^+^/CD4^+^ T cells (particularly CD107a^+^ cytotoxic T cells) [148].

### Radiopharmaceutical therapy and cytokine therapy

**Fig. 6:**
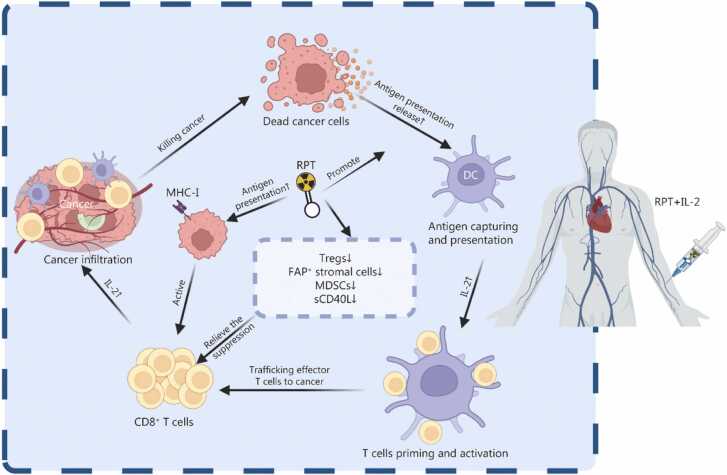
Synergistic immunomodulation of the tumor microenvironment by combined radiopharmaceutical therapy (RPT) and interleukin-2 (IL-2) treatment. The combination of RPT and IL-2 orchestrates enhanced anti-tumor immunity through complementary mechanisms. In the cancer immunity cycle, dying cancer cells release antigens that are captured by APCs and DCs, where IL-2 potentiates DC-mediated antigen presentation to effector T cells, driving adaptive immune responses. IL-2 further promotes the infiltration and cytotoxic function of activated T cells, which eliminate cancer cells and perpetuate the immunogenic cycle through subsequent antigen release. RPT synergistically enhances this process by 1) inducing ICD to amplify tumor antigen availability, while upregulating major histocompatibility complex class I (MHC-I) and co-stimulatory molecules to boost T cell priming; and 2) remodeling the immunosuppressive TME via depletion of FAP⁺ stromal cells, MDSCs, and Tregs, alongside reducing inhibitory factors like sCD40L. Together, these interventions establish a self-reinforcing loop, where RPT provides antigenic fuel and tempers immune suppression, while IL-2 magnifies T cell activation and effector functions, culminating in sustained tumor control. TME. Tumor microenvironment; APCs. Antigen-presenting cells; DCs. Dendritic cells; ICD. Immunogenic cell death; FAP. Fibroblast activation protein; MDSCs. Myeloid-derived suppressor cells; Tregs. Regulatory T cells; sCD40L. Soluble CD40 ligand.

Cytokines play a crucial role in regulating immune responses and cellular behaviors, including ILs, IFNs, TNF, chemokines, and growth factors. Cytokine therapy regulates intercellular communication within the TME, suppresses tumor growth, and enhances the efficacy of cancer RPT [149]. In breast and colon cancers, ^225^Ac-labeled M5A targets CEA^+^ tumors, releasing antigens, reducing Tregs, and priming a pro-immunogenic TME. M5A-IL-2 then boosts CD8^+^ T cells and increases the CD8^+^/Treg ratio. This sequential approach, targeted α-particle therapy, removes immunosuppression and is combined with IL-2 to enhance T-cell activity. The TME acts as a bridge, amplifying radiation-induced immunogenicity while accelerating IL-2-driven anti-tumor immunity [150] (Fig. 6). Similarly, in another study, RPT specifically targets fibroblast activation protein (FAP)^+^ stromal cells, inducing localized radiation damage that leads to tumor cell death and antigen release; meanwhile, L19-IL-2 exploits its tumor neovasculature-targeting property to precisely deliver IL-2 within the TME, activating infiltrated CD8⁺ T cells and NK cells while promoting myeloid cell polarization toward a proinflammatory phenotype. Proteomic analysis confirms that the combined treatment significantly upregulates inflammatory factors such as IFN-γ and downregulates immunosuppressive cytokines within the TME, effectively converting immunologically “cold” tumors into “hot” tumors. This dynamic remodeling overcomes intrinsic immunosuppressive barriers in the TME, enabling sustained anti-tumor responses via endogenous immune activation while maintaining low radiation doses [151].

### Diagnostic radiopharmaceutical therapy and immune checkpoint inhibitors

Traditional imaging has limitations in evaluating IT response; new radionuclide diagnostics technologies based on multi-component collaborative tracing of the TME are revolutionizing precision cancer diagnosis [152], [153]. ^89^Zr-labeled anti-PD-L1 monoclonal antibodies used in neck cancer and melanoma immune PET/CT, not only decode the dynamics of intrinsic PD-L1 in tumor cells but also non-invasively reveal the spatiotemporal evolution patterns of immune checkpoint proteins within the TME [154]. The ^99m^Tc NM-01 tracer can dynamically capture the expression of PD-L1 in TME-critical effector cells. Its non-competitive tracing properties enable longitudinal monitoring of immune biomarker fluctuations in the microenvironment during anti-PD-(L)1 therapies [155]. [^18^F] AlF-FAPI-74 PET imaging allows real-time tracking of FAP expression dynamics in cancer-associated fibroblasts (CAFs), unveiling molecular trajectories of TME stromal remodeling post-CAR-T cell therapy [156]. ^89^Zr-oxine-labeled CAR T cells retained key biological functions, including viability, proliferation, chemotaxis, cytotoxicity, and specificity for interleukin-13 receptor alpha 2 (IL-13Rα2)-positive tumor cells, enabling non-invasive PET imaging to monitor CAR T cell dynamics [157]. Clinical trials on the application of diagnostic RPTs in cancer are summarized in Table 2 [158], [159], [160], [161], [162], [163], [164], [165], [166], [167], [168], [169].

**Table 2:** Clinical trials of diagnostic radiopharmaceutical therapies (RPTs) in cancer.

**Cancer types**	**Diagnostic RPTs**	**ID**	**Status**	**No. of patients**	**Stage**	**References**
Solid tumor	^68^Ga-THP-APN09	NCT05156515	Recruiting	20	NA	[158]
Solid tumor	^68^Ga-NODAGA-SNA006	NCT05126927	Completed	11	Early phase 1	[159]
Advanced or metastatic malignancies	^89^Zr crefmirlimab berdoxam	NCT05013099	Active, not recruiting	70	Phase II	[160]
PD-L1^+^ solid tumors	^89^Zr-labeled KN035	NCT04977128	Unknown status	20	NA	[161]
Hematopoietic and lymphoid cell neoplasm	^64^Cu-DOTA-pembrolizumab	NCT04605614	Withdrawn	0	Phase I	[162]
Esophageal and rectal cancer	^89^Zr-atezolizumab	NCT04564482	Recruiting	20	NA	[163]
Non-small cell lung (NSCLC)	^68^Ga-NOTA-WL12	NCT04304066	Completed	50	NA	[164]
Advanced PD-L1^+^ malignancies	^89^Zr-DFO-REGN3504	NCT03746704	Terminated	2	Phase I	[165]
Metastatic melanoma	^89^Zirconium-labeled ipilimumab	NCT03313323	Unknown status	29	Phase II	[166]
Solid malignancies or Hodgkin’s lymphoma	^89^Zr-Df-IAB22M2C	NCT03107663	Completed	15	Phase I	[167]
Advanced or metastatic melanoma or NSCLC	^89^Zr-pembrolizumab	NCT02760225	Completed	18	NA	[168]
NSCLC	^89^Zr-pembrolizumab	NCT03065764	Unknown status	10	Phase II	[169]

PD-L1. Programmed cell death ligand 1; Ga. Gallium; PET. Positron emission tomography; CT. Computed tomography; NA. No data; Zr. Zirconium; Cu. Copper

## Clinical studies of radiopharmaceutical therapy combined with immunotherapy

The combination of RPT and IT is being increasingly studied, with expanding clinical research across diverse malignancies that progressively uncovers both synergistic effects and treatment limitations. Presented below are clinical data from studies in prostate cancer, hepatocellular carcinoma (HCC), neuroendocrine tumors, and colorectal cancer, demonstrating advancements in this emerging field and providing actionable insights for refining combination strategies (Table 3) [148], [170], [171], [172], [173], [174], [175], [176], [177], [178], [179], [180], [181], [182], [183], [184].

**Table 3:** The therapeutic effect of radiopharmaceutical therapy (RPT) combined with IT in cancers.

**RPT**	**IT**	**Study type/ID/Patients**	**Indication**	**Phase**	**Therapeutic outcomes**	**References**
^177^Lu-PSMA-617	Pembrolizumab	Clinical trials/NCT03658447	mCRPC patients with high PSMA expression	Phase I	The combination of ^177^Lu-PSMA-617 and pembrolizumab had promising activity. Toxicities were generally consistent with those of single-agent ^177^Lu-PSMA-617 and pembrolizumab and were not clearly augmented by combination use	[170]
^177^Lu-PSMA-617	Pembrolizumab	Clinical trials/NCT03805594	mCRPC	Phase Ib study	^177^Lu-PSMA-617 followed by pembrolizumab maintenance, demonstrated safety and encouraging preliminary activity, with a confirmed PSA50 response rate of 56%	[171]
^177^Lu-PSMA-617	Ipilimumab and nivolumab	Clinical trials/NCT05150236	mCRPC	Phase II	Combination therapy improved 12-month PSA progression-free survival (33% vs. 17%), but grade 3–4 adverse events occurred in 75% (including 7% myocarditis); the trial was terminated early due to safety concerns	[172]
^177^Lu-PSMA-617	Pembrolizumab	Case/2 patients	mCRPC	NA	The combination therapy was well-tolerated, without significant hematologic toxicity, and led to both radiographic and biochemical responses	[173]
^177^Lu-PSMA-617	Nivolumab	Case/1 patient	Advanced uterine leiomyosarcoma	NA	Show good tolerability and a significantly reduced tumor growth rate	[174]
^177^Lu-DOTA^0^-Tyr^3^-octreotate (Lutathera)	Nivolumab (ICI)	Clinical trials/NCT03325816	Neuroendocrine tumors of the lung	Phase I	Lutathera plus nivolumab was well tolerated and showed signs of anti-tumor activity	[175]
^177^Lu-DOTATOC PRRT	Avelumab and the ipilimumab/nivolumab	Case/1 patient	Metastatic Merkel cell carcinoma	NA	Achieved rapid and marked regression of bone metastases, maintaining a durable partial response for ≥5 months	[176]
^177^Lu-DOTATOC PRRT	Ipilimumab plus nivolumab	Case/1 patient	Temozolomide-resistant pituitary carcinoma	NA	Achieved sustained disease control for 3.5 years with PRRT followed by ipilimumab plus nivolumab, demonstrating a 61% reduction in target lesion volume	[177]
^153^Sm-EDTMP	PSA-TRICOM	Clinical trials/NCT00450619	mCRPC	Phase II	Median PFS was 1.7 months vs. 3.7 months (combination arm; *P*=0.041, *HR*=0.51). No ≥30% PSA decline occurred with ^153^Sm-EDTMP alone, whereas 4/21 (19%) in the combination arm had ≥30% decline (3 with ≥50% decline). Toxicity was similar and dose-dependent	[148]
^223^RaCl_2_	Sipuleucel-T (cancer vaccine)	Clinical trials/NCT02463799	Asymptomatic bone mCRPC	Phase II	The combination group had higher rates of >50% PSA decline, longer PFS (39 weeks vs. 12 weeks), and improved OS (not reached vs. 2.6 years)	[178]
^223^RaCl_2_	Atezolizumab (anti-PD-L1)	Clinical trials/NCT02814669	mCRPC	Phase Ib study	The combinations demonstrated greater toxicity than either drug alone, with no clear evidence of additional clinical benefit	[179]
Y-90 radioembolization	Nivolumab (ICI)	Clinical trials/NCT03033446	HCC	Phase II	^90^Y radioembolization followed by nivolumab resulted in an encouraging objective response rate in patients with advanced hepatocellular carcinoma	[180]
Y-90 radioembolization	Nivolumab	Case/1 patient	Advanced HCC	NA	The combination exhibited significant shrinkage of target lesions on follow-up imaging after receiving Y-90 radioembolization combined with nivolumab	[181]
Y-90 radioembolization	Nivolumab or ipilimumab	Single-center retrospective cohort study/26 patients	Advanced HCC	NA	The combination therapy showed marked efficacy, with a 17.2-month median OS and 77% ORR (27% CR) in target lesions, significantly outperforming Y-90 radioembolization monotherapy historically	[182]
Y-90 radioembolization	Durvalumab and tremelimumab	Clinical trials/NCT03005002	Microsatellite stable colorectal cancer liver metastases	Phase II	Y90 radioembolization can be added safely to durvalumab and tremelimumab, but did not promote tumor‐directed immune responses	[183]
Yttrium-90 transarterial radioembolization	CTLA-4/PD-1 inhibitors	Retrospective analysis of 11 patients	Uveal melanoma liver metastases	NA	Achieved a disease control rate of 63.6%, which was significantly superior to historical controls	[184]

Lu. Lutetium; ICI. Immune checkpoint inhibitor; Ra. Radium; Y. Yttrium; Sm. Samarium; mCRPC. Metastatic castration-resistant prostate cancer; HCC. Hepatocellular carcinoma; NA. No data; PSMA. Prostate-specific membrane antigen; IT. Immunotherapy; PSA. Prostate-specific antigen; PFS. Progression-free survival; OS. Overall survival; ORR. Objective response rate; PRRT. Peptide receptor radionuclide therapy

These studies collectively demonstrate the potential of combining RPT with IT across various cancer types, although a minority show variability in results; further research is needed to optimize these combination approaches. Several clinical trials with combined RPT and IT are currently ongoing (Table 4) [185], [186], [187], [188], [189], [190], [191], [192], [193], [194], [195], [196], [197], [198], [199], [200], [201].

**Table 4:** Ongoing clinical trials of radiopharmaceutical therapy (RPT) combination with immunotherapy in cancer.

**Cancer types**	**RPT**	**IT**	**ID**	**Status**	**No. patient**	**Stage**	**References**
MCC	^177^Lu-DOTA-TATE	Pembrolizumab (ICI)	NCT05583708	Suspended	18	Phase II	[185]
Renal cancer	^177^Lu-Gerentuximab	Nivolumab (ICI)	NCT05239533	Recruiting	41	Phase II	[186]
mCRPC	^177^Lu-PSMA-617	Ipilimumab and nivolumab (ICI)	NCT05150236	Active, not recruiting	93	Phase II	[187]
SCLC	^177^Lu-DOTA-TATE	Tislelizumab (ICI)	NCT05142696	Recruiting	200	Phase I/II	[188]
mCRPC	^225^Ac-J591	Pembrolizumab (ICI)	NCT04946370	Recruiting	76	Phase I/II	[189]
NETs	^177^Lu-DOTA-TATE	Nivolumab (ICI)	NCT04525638	Unknown status	30	Phase II	[190]
MCC	^177^Lu-DOTA-TATE	Avelumab (ICI)	NCT04261855	Recruiting	38	Phase I/II	[191]
mCRPC	^223^RaCl_2_	Nivolumab (ICI)	NCT04109729	Recruiting	36	Phase I/II	[192]
Metastatic colorectal cancer	^90^Y radioembolization	Durvalumab (ICI)	NCT04108481	Suspended	18	Phase I/II	[193]
mCRPC	^223^RaCl_2_	Avelumab (ICI)	NCT04071236	Recruiting	90	Phase I/II	[194]
NSCLC with bone metastases	^223^RaCl_2_	Pembrolizumab (ICI)	NCT03996473	Terminated	164	Phase I/II	[195]
Hepatic metastases NET neuroendocrine tumor	^177^Lu-DOTA-TATE	Pembrolizumab (ICI)	NCT03457948	Active, not recruiting	32	Phase II	[196]
Recurrent/metastatic thyroid cancers	^131^I	Durvalumab (ICI)	NCT03215095	Active, not recruiting	11	Phase I	[197]
Hepatocellular carcinoma	^90^Y radioembolization	Pembrolizumab (ICI)	NCT03099564	Active, not recruiting	30	Phase I	[198]
Relapsed/refractory pediatric neuroblastoma	^131^I-meta-iodobenzylguanidine	Nivolumab (ICI)	NCT02914405	Recruiting	44	Phase I	[199]
Uveal melanoma with liver metastases	^90^Y radioembolization	Ipilimumab and Nivolumab (ICI)	NCT02913417	Recruiting	26	Phase I/II	[200]
Uveal melanoma with liver metastases	^90^Y glass microspheres	Ipilimumab (ICI)	NCT01730157	Terminated	6	Early phase 1	[201]

Lu. Lutetium; ICI. Immune checkpoint inhibitor; Ac. Actinium; Y. Yttrium; SCLC. Small cell lung cancer; mCRPC. Metastatic castration-resistant prostate cancer; Ra. Radium; I. Iodine; MCC. Merkel cell carcinoma; DOTA. 1,4,7,10-tetraazacyclododecane-1,4,7,10-tetraacetic acid; TATE. Tyr³-octreotate; ICI. Immune checkpoint inhibitor; NETs. Neuroendocrine tumours

## Discussion and prospects

**Fig. 7:**
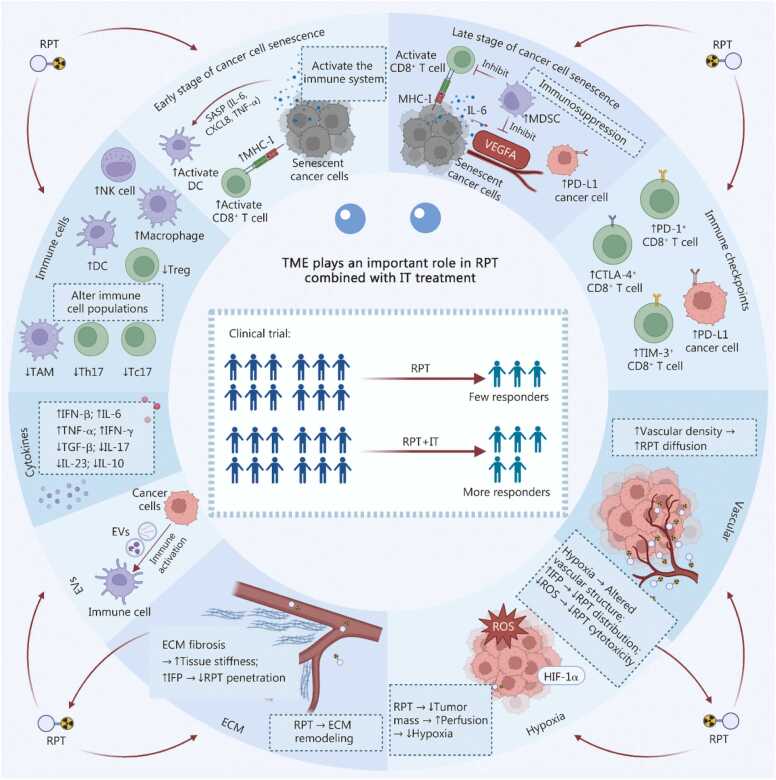
The TME plays a bridging role in the synergistic enhancement of RPT and IT. RPT reshapes the TME into an immunologically active state, thus providing targets and synergy for IT. Moreover, the TME features like vascular distribution, hypoxia, ECM density, and immunosuppressive components collectively regulate drug delivery and efficacy, while good vasculature aids drug transport. Clinical trials of RPT combined with IT have demonstrated survival benefits. RPT. Radiopharmaceutical therapy; IT. Immunotherapy; ROS. Reactive oxygen species; ECM. Extracellular matrix; IL. Interleukin; TGF. Transforming growth factor; SASP. Senescence-associated secretory phenotype; CXCL. C-X-C motif chemokine ligand; DCs. Dendritic cells; PD-L1. Programmed cell death ligand 1; MDSCs. Myeloid-derived suppressor cells; MHC. Major histocompatibility complex; VEGFA. Vascular endothelial growth factor A; IFN. Interferon; TNF. Tumor necrosis factor; EVs. Extracellular vesicles; NK cells. Natural killer cells; Tregs. Regulatory T cells; TAM. Tumor-associated macrophage; Th. T helper cells; Tc. Cytotoxic T cells; CTLA. Cytotoxic T-lymphocyte-associated protein; TIM. T-cell immunoglobulin and mucin domain-containing protein; HIF. Hypoxia-inducible factor; IFP. Interstitial fluid pressure.

This review methodically elucidates the interactions between RPT and components of the TME. RPT promotes anti-tumor effects primarily through the induction of tumor cell senescence. However, the efficacy of RPT can be compromised by factors such as the hypoxic microenvironment, aberrant vascularization, and the presence of immunosuppressive elements, including Tregs and TAMs. RPT has been shown to reprogram the TME into an immunostimulatory state by modulating immune cell subsets and releasing immunogenic signals. When combined with IT, RPT synergistically transforms the TME, enhancing anti-tumor immunity and reducing immune suppression, which leads to improved tumor responses, decreased metastasis, and heightened treatment efficacy. Clinical trials, including those involving ^177^Lu-PSMA-617 combined with pembrolizumab and ^177^Lu-DOTA^0^-Tyr^3^-octreotate with nivolumab, have demonstrated clinically significant enhancements in PFS. Fig. 7 summarizes the main content of this study. These clinical studies also underscore the challenges related to treatment-induced toxicities and varied response patterns [170], [179]. Future research should aim to precisely modulate the TME and refine combination strategies to maximize efficacy and patient outcomes. Given the critical role of the TME in the effectiveness of both RPT and IT, we propose the following potential strategies for TME remodeling to augment the effectiveness of RPT and its integration with IT.

### Targeting cancer-associated fibroblasts

Strategies targeting CAFs mainly include direct depletion of CAFs and interference with CAF-derived effector molecules. Among them, FAP-targeted radioligands, such as ^177^Lu-FAP-2286/2287, can directly deplete tumor-promoting CAFs, reduce extracellular matrix-mediated physical barriers, and facilitate immune cell infiltration within the tumor microenvironment. However, this approach may not be uniformly beneficial, because irradiated CAFs can also increase the secretion of stromal cell-derived factor 1 (SDF-1)/CXCL12, which activates the C-X-C motif chemokine receptor 4-p38 pathway and thereby promotes tumor cell migration, invasion, EMT, and radioresistance [202]. This contradiction reflects the dynamic nature and complexity of the TME, which can be reconciled through combined strategies based on existing research. For instance, combining PD-L1 inhibitors with the SDF-1 antagonist AMD3100 counteracts this resistance [202]. CAF-secreted IL-6 promotes immune evasion, and the IL-6 inhibitor SOM230 (Pasireotide) enhances chemotherapy/IT sensitivity in pancreatic cancer [202]. Additionally, targeting CAFs via RPT exerts a synergistic effect with ICIs by enhancing CD8⁺ T cell infiltration and type I IFN responses [202]. Targeting CAFs with RPT is a promising theranostic strategy for cancer, owing largely to the selective expression of FAP on CAFs and its minimal expression in most normal tissues [203]. FAP-specific radiotracers (e.g., FAPI-C12/C16 [204], ^177^Lu-DOTA-2P(FAPI)2 [205]) optimize tumor-to-background ratios, improving RT precision and therapeutic efficacy [206]. Albumin-bound FAP tracers enhance tumor uptake/retention [205], while radionuclide choice (α-emitters like ^225^Ac for CAF-dominant niches vs. β-emitters like ^177^Lu for large clusters) guides personalized delivery [207]. In addition to depleting CAFs, concurrent targeting of the TME’s vascular system has been proposed to synergistically enhance therapeutic efficacy [208]. A dual strategy is employed to address the physical barriers constituted by CAFs and collagen. On one hand, negatively charged NPs are used to circumvent CAF entrapment [208]. On the other hand, CAF-depleting agents such as focal adhesion kinase inhibitors are applied to reduce collagen deposition, which potentially increases the drug diffusion coefficient 10-fold. To tackle hyperdense microvasculature, anti-angiogenic drugs such as bevacizumab are employed to reduce vascular density by 50%, significantly curtailing drug drainage and preventing rapid drug efflux [208].

Targeted FAP radioligands like ^177^Lu-FAP-2286 are advancing clinically with high translational potential. Other agents, such as FAPI-C12/C16 and various inhibitors, remain largely in preclinical stages, requiring further validation of efficacy and translation pathways. Overall, targeting CAFs, especially via FAP-based tracers, holds strong translational promise.

### Improving tumor hypoxia and regulating angiogenesis

Inhibiting tumor angiogenesis, which is driven by vascular endothelial growth factor/vascular endothelial growth factor receptor (VEGF/VEGFR) signaling or overexpression of integrin αvβ3, can enhance the delivery of RPT drugs. Concurrently, targeting VEGF/VEGFR also establishes a basis for precise targeted imaging utilizing RPT. Radioligands, such as ^68^Ga/^64^Cu/^99m^Tc-labeled agents and [^125^I]p-NPAM, exploit αvβ3/VEGFR targets to augment tumor-to-background ratios [209], [210]. Furthermore, CD13/angiogenesis-associated aminopeptidase N-targeted asparagine-glycine-arginine peptides and CD105-directed ^177^Lu-TRC105 inhibit angiogenesis and have been shown to improve survival in preclinical models [209], [210]. Challenges in vascular off-target damage are mitigated by combining RPT with anti-VEGFR2 antibodies or anginex to enhance tumor perfusion and drug delivery [211]. Hypoxia-driven resistance is addressed through hypoxia-activated prodrugs and imaging agents like ^18^F-FMISO/^64^Cu-diacetyl-bis(N_4_-methylthiosemicarbazone) (ATSM) that selectively accumulate in low-oxygen regions [212]. Clustered regularly interspaced short palindromic repeats **(**CRISPR)/Cas-mediated knockout of hypoxia-inducible factor 1α (HIF-1α)/glucose transporter type 1 (GLUT-1) disrupts hypoxia adaptation, while vascular normalization strategies improve oxygenation and radiosensitivity [212].

Anti-angiogenic drugs represented by bevacizumab have been widely used in clinical practice and hold high translational potential when combined with RPT. In contrast, hypoxia-activated prodrugs, vascular normalization, and CRISPR editing remain largely preclinical, requiring further validation of safety and efficacy.

### Modulating the extracellular matrix barrier

Elevated levels of ECM components, such as collagen, fibronectin, and HA, exacerbate intratumoral pressure and hypoxia, thereby promoting radioresistance [213], [214]. Strategies to remodel the ECM include hyaluronidase (pegvorhyaluronidase alfa) and losartan, which reduce the rigidity of HA and collagen, improve vascular perfusion, enhance T-cell infiltration, and synergize with RT or IT [215], as well as small molecules such as 6-Diazo-5-oxo-L-norleucine and AZD1480 (an investigational JAK2 inhibitor), which further inhibit ECM synthesis and disrupt collagen architecture, thereby amplifying anti-PD-1 efficacy and tumor regression [216]. Treatment with hymecromone (4-methylumbelliferone) significantly reduces HA accumulation in various murine tumor models [217]. Additionally, another study shows that intratumoral application of hyaluronidase improved response to PD-L1-directed IT [218].

Currently, hyaluronidases (such as pegvorhyaluronidase alfa) have been approved by the Food and Drug Administration for specific cancers, and losartan is an already marketed drug; their clinical translational potential in combination with RT/IT is relatively high. Drugs such as 6-Diazo-5-oxo-L-norleucine and AZD1480 have been primarily validated in preclinical models, with their human efficacy, safety, and combination synergies still at the proof-of-concept stage, requiring further translational development.

### Metabolic reprogramming interventions

Metabolic reprogramming is an emerging field in cancer therapy. Similar to cancer cells, TAMs rely on glycolysis to sustain their rapid proliferation. Mannose inhibits glycolysis and promotes PD-L1 degradation, limiting the growth of both TAMs and cancer cells [219]. Levamisole hydrochloride recruits lysosomes within TAMs, enhancing the metabolic inhibitory effects of mannose by degrading mannose-6-phosphate (M6P) isomerase, inhibiting glycolysis, and inducing autophagy in cancer cells and TAMs [220]. A biocompatible liposome (M/LM-Lipo) co-encapsulating mannose and levamisole hydrochloride, combined with RT in a 4T1 mouse model, demonstrates significant therapeutic efficacy by reducing cancer cells and immunosuppressive M2 macrophages through metabolic interference [221]. In the context of RT-induced hypoxia, which upregulates PD-L1 on MDSCs and promotes lactate-mediated MDSC activation, hafnium-based metal-organic framework NPs (Hf-MOL) reduce lactate levels by delivering ROS, improving hypoxia in the TME [222]. The novel strategy of combining RT with radiodynamic therapy using low-dose RT and either lactate dehydrogenase A inhibition or Hf-MOL-mediated ROS delivery is a safe and effective approach to indirectly reduce MDSC numbers by modulating the TME [223].

Mannose, levamisole, and nanodelivery systems show preclinical evidence for tumor metabolic modulation and RT synergy, but face substantial barriers to clinical translation due to challenges in delivery efficiency, targeting specificity, and long-term safety.

### Remodeling tumor microenvironment with new technology

Compared to the traditional approach of directly injecting DCs or Toll-like receptor (TLR) agonists into tumors, NP-based DC-targeting systems offer a more efficient antigen delivery platform with superior therapeutic outcomes and lower toxicity [224]. Bioactive polysaccharide NPs not only facilitate DC maturation by regulating the nuclear factor κB (NF-κB) signaling pathway through TLRs (notably TLR4) and upregulating co-stimulatory molecules such as CD40, CD80, and CD86, but also amplify the abscopal effect of RT through the remodeling of the immunosuppressive TME [225]. In nanotherapy, multiple strategies are employed to reduce TAMs or drive M1 polarization. For example, Zirconium-gadolinium nanorods (ZGd NRs) induce ICD via X-ray deposition while depleting TAMs, demonstrating significant synergy in 4T1 breast cancer metastasis and CT26 models [226]. NPs loaded with TLR7/8 agonists such as cyclodextrin nanoparticles (CDNPs), Smac-TLR7/8 hydrogels, TLR9 agonist CpG-conjugated gold NPs (CpG@Au NPs), and multifunctional NPs composed of polylysine, iron oxide, and CpGs, effectively induce M2-to-M1 macrophage polarization following RT [227]. Chen *et al*. [228] developed a multifunctional nanoscavenger combining catalase, chymotrypsin (CHY), calcium peroxide NPs, and photosensitizer chlorin e6 with CLT1 peptide for fibronectin targeting. CHY-ROS synergy disrupts the ECM, enhancing penetration. Calcium peroxide NPs produce Ca²⁺ and H₂O₂ for calcium therapy, while catalase converts H₂O₂ to oxygen, alleviating hypoxia.

The integration of CRISPR technology with microfluidics has shown considerable promise in modulating the TME, facilitating precise functional interventions in immune and stromal cells surrounding tumors through gene editing [229]. Specifically, CRISPR can be employed to target and regulate essential signaling molecules within the TME, such as IL-10 and TGF-β, and indirectly modifies the immunosuppressive TME by degrading specific miRNAs or mRNAs through the enzymatic activity of Cas12a/13a [229]. Furthermore, utilizing its capability for single-cell manipulation, microfluidic chips can accurately isolate and edit circulating tumor cells or TAMs within the TME, such as reprogramming TAMs from an M2 to an M1 phenotype, thereby enhancing anti-tumor immune responses [229]. Nevertheless, the potential off-target effects of CRISPR in complex TME might affect healthy cells, and the challenges posed by tumor heterogeneity and the sample processing requirements for integrating microfluidic platforms should be addressed. Future efforts should concentrate on enhancing the targeting specificity of CRISPR and fostering device standardization to expedite the clinical application of this technology in TME modulation and precision therapy.

While nanotechnology and CRISPR/microfluidics show promising efficacy and multifunctionality in animal models, they remain at proof-of-concept or preclinical stages. Their translation faces challenges in delivery, stability, safety, and scalable production, requiring sustained interdisciplinary efforts.

### Remodeling tumor microenvironment by targeting nerve-immune-cancer interactions

In the past decade, growing evidence highlights the critical role of nerve-cancer and nerve-immune interactions in tumor progression and treatment response. Both the central nervous system (CNS) and peripheral nervous system (PNS) participate in modulating TME and anti-tumor immunity [230], [231], [232], [233].

The CNS regulates non-CNS tumor immunity mainly via the hypothalamic-pituitary-adrenal axis and autonomic nervous system (including nerves and hormones/neurotransmitters), which results in abnormal neurotrophic signaling in the TME, dysregulation of both systemic/local inflammation and oncoimmunological functions, thereby inducing the proliferation, invasion, and angiogenesis of cancer [231], [234], [235]. For instance, psychological stress promotes the remodeling of the TME toward an immunosuppressive state by, on one hand, elevating glucocorticoid levels, inhibiting type I IFN responses in DCs, and impairing IFN-γ^+^ T cell activation, and on the other hand, activating β-adrenergic receptors and their downstream signaling pathways [e.g., dopamine receptor D2 (DRD2)/ extracellular signal-regulated kinase (ERK)/β-catenin]. Drugs targeting stress-related pathway receptors have been shown to exert anti-tumor effects [236], [237], [238], [239]. In contrast, reward system activation suppresses MDSC-mediated immunosuppression by reducing sympathetic innervation and noradrenergic input to the bone marrow, thereby exerting a protective anti-tumor immune effect [240]. Meanwhile, β-blockers (e.g., propranolol) inhibit tumor growth by suppressing proinflammatory factors (TNF-α/IL-6) and angiogenesis (VEGF/Akt), and their combination with ICIs (anti-CTLA-4/PD-1) has demonstrated synergistic efficacy [241]. Furthermore, psychological interventions (e.g., cognitive behavioral therapy) targeting stress-related neuroendocrine pathways (such as the hypothalamic-pituitary-adrenal axis) have also been proven to improve patient outcomes [242].

The PNS, including sympathetic, parasympathetic, and sensory nerves, interacts directly with cancer, stromal, and immune cells to promote tumorigenesis, mainly via two pathological processes, perineural invasion (PNI) and neurogenesis/innervation [40], [41], [243]. In PNI, endoneurial macrophages secrete glial cell line-derived neurotrophic factor (GDNF), attracting and activating cancer cell migration toward nerve cancer cells via rearranged during transfection signaling/GDNF family receptor alpha 1 [244]. Dedifferentiated Schwann cells from nerves also promote PNI by recruiting inflammatory monocytes/macrophages through C-C motif chemokine ligand 2/C-C motif chemokine receptor 2, thereby degrading the protective perineurium [245].

In neurogenesis/innervation, sympathetic nervous system activation leads to the release of norepinephrine, which acts on β-adrenergic receptors expressed by various immune cells in the TME. This signaling enhances Treg and MDSC expansion while suppressing the infiltration and function of CTLs and DCs [246], [247], [248], with effects reversible by β-blockers [249]. In contrast, cholinergic signaling via the vagus nerve reduces CD11b^+^ myeloid cells and TNF-α in the TME, indirectly inhibiting tumor growth and metastasis by modulating immune cell composition and inflammatory responses [250]. In addition, computational models reveal that mechanical stresses in solid tumors (such as solid stress and IFP) may influence nerve-immune-cancer interactions through complex biophysical mechanisms [251]. Specifically, solid stress not only creates a physical barrier that restricts immune cell migration but also reshapes the immunosuppressive TME via hypoxia [251]. Additionally, direct mechanical compression of neurons exacerbates local inflammatory response imbalance [251].

Although RPT or RPT-IT remains underexplored in the context of nerve-cancer or nerve-immune-cancer interactions, conventional RT has shown dual effects. For PNI, RT impairs PNI by modulating the nerve microenvironment, such as reducing GDNF secretion, which is independent of direct cytotoxicity to cancer cells [252]. As mentioned above, endoneurial macrophage is a main source of GDNF [244]. RT selectively improves survival in node-negative esophageal cancer patients with PNI (PNI-positive), but not in those without PNI [253]. For neurogenesis/innervation, however, short-course radiation therapy can enhance adrenergic-driven tumor innervation, mitigated by β-blockers [254]. There is robust evidence that conventional RT can cause peripheral neuropathy at high doses [255], [256], whereas RPT is more TME-targeted with lower neuropathy risk, highlighting the unmet need to investigate RPT’s unique influence on nerve-immune-tumor crosstalk.

RT and neuromodulation evidence suggest RPT’s targeted delivery and confined radiation may enable precise modulation of tumor-innervated areas while sparing healthy nerves, potentially lowering neurotoxicity [257]. This localized exposure may regulate tumor-associated neurons, immune cells, and cancer cells, reshaping the immunosuppressive TME [58], [59]. We hypothesize that RPT, especially combined with neuromodulators or IT, may synergistically enhance anti-tumor effects through localized modulation of the neuro-immune-tumor unit. Future studies should further elucidate the role of RPT and RPT-IT in nerve cancer, especially nerve-immune-cancer interactions. Meanwhile, it is necessary to conduct randomized controlled trials to validate the combination effects of RPT and IT, especially in conjunction with neuromodulatory agents like β-blockers. This will provide mechanistic insight and translational value, enhancing RPT’s efficacy and its synergistic effects with IT and expanding its clinical application.

### Challenges for radiopharmaceutical therapy-immunotherapy combined therapies

Combined RPT and IT shows therapeutic potential but faces complex challenges [258]. Clinically, RPT is better tolerated than chemotherapy, with fewer severe adverse events [16]. Common toxicities include hematologic toxicities [16], such as thrombocytopenia, neutropenia, anemia, and myelosuppression [259]. In PSMA-targeted therapy, xerostomia is frequently observed due to PSMA expression in the salivary glands, while nephrotoxicity may arise from renal excretion [260]. Additionally, specific targets may cause toxicity in other organs [such as pancreatic effects in gastrin-releasing peptide receptor (GRPR)-targeted therapy] [261]. Secondary myelodysplastic syndromes have been reported with some radioimmunotherapy approaches [262]. Combined with ITs, toxicities are additive, requiring management of both RPT-related effects and immune-related adverse events (e.g., gastrointestinal, pulmonary, endocrine) [172], [263]. Although the tumor-selective distribution of RPT theoretically may help reduce unnecessary irradiation of circulating immune cells, this does not lower the overall toxicity risk of the combination therapy [16].

The combination of RPT and IT also faces mechanistic challenges within the TME. First, while killing tumor cells, RPT can induce negative immune regulation, such as increasing immunosuppressive Tregs or upregulating PD-1/CTLA-4 on effector T cells, potentially leading to exhaustion and reduced IT synergy [82]. Second, dose-limiting myelosuppression caused by RPT directly impairs immune system function. This may counteract the benefits of IT [47]. Additionally, radiation-induced immunogenic signals, such as the release of HMGB1 and ATP within the TME, if not properly synergized with IT, might activate compensatory immunosuppressive pathways, thus promoting tumor adaptive tolerance [264]. The post-treatment evolution of the TME, characterized by CAF activation and an increase in immunosuppressive exosomes, may contribute to acquired resistance, necessitating innovative combination strategies that target TME remodeling [265]. Consequently, understanding the impact of these dynamic changes on the response to combination therapy is essential for devising methods to counteract treatment resistance.

The following factors collectively hinder optimal combination strategies. The ideal timing and sequence of RPT with IT are undefined; inappropriate regimens may prevent synergy or cause antagonism, partly explaining inconsistent preclinical results [47]. Patient selection is also critical, as prior treatments (e.g., chemotherapy) can raise hematologic toxicity risk with RPT [266], while features such as a high bone metastasis burden are associated with a greater risk of myelosuppression [267]. The heterogeneity of TME can influence the synergistic effects of RPT and IT [268]. For instance, variability in the TME, such as the uneven expression of PSMA or somatostatin receptors, can restrict the effectiveness of the combination therapy, thereby preventing some patients from achieving optimal outcomes [269], [270]. Therefore, investigating TME-related predictive biomarkers may help identify potential responders to RPT/IT combination therapy [271]. However, while PD-L1 expression in the TME guides ICI use [272] and tumor target expression is essential for RPT [273], their predictive value for RPT/ICI combinations requires further validation [274]. Currently, few studies explore how varying RPT dosages differentially regulate radiobiological effects via TME modulation [275], [276]. Although high-activity and low-activity RPT potentially remodel TME immune landscapes via distinct cell death pathways [277], such TME response mechanisms demand experimental confirmation. Research on the impact of circadian rhythms on the TME and cancer therapy has revealed that circadian rhythms regulate immune cell migration, cytokine secretion, and endothelial permeability within the TME, further highlighting the necessity of time-sequenced interventions to enhance therapeutic efficacy [278], [279], [280]. However, a few studies suggest that concurrent administration may be superior to sequential administration [88], [177]. Prolonged low-dose-rate irradiation may impair infiltrating immune cells [281], [282].

**Fig. 8:**
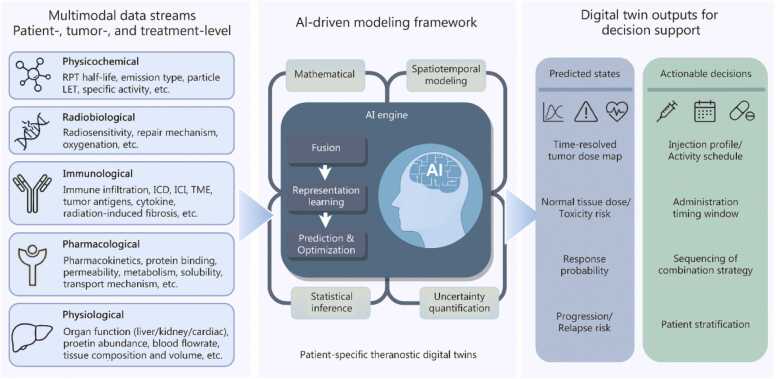
Framework for patient-specific theranostic digital twins based on AI-driven integration of multimodal data. Multimodal data are needed at patient-, tumor-, and treatment-level resolution, including physicochemical, radiobiological, immunological, pharmacological, and physiological domains. These data will be processed and analyzed through advanced AI, and then will be used to predict the states and optimize the decision support. AI. Artificial Intelligence; LET. Linear energy transfer; ICD. Immunogenic cell death; ICI. Immune checkpoint inhibitor; TME. Tumor microenvironment.

To address TME heterogeneity and dynamics, emerging technologies such as single-cell sequencing, spatial transcriptomics, and multimodal theranostics are urgently needed [283]. The field is shifting from “theranostics” toward an intelligent closed-loop system of “theranostics-regeneration integration”, with artificial intelligence (AI) as the key driver [283]. AI analyzes multi-omics data and simulates interactions between therapeutic carriers (e.g., nanomaterials) and biological systems to predict and optimize delivery efficiency, stimulus-response thresholds, and biodegradation kinetics [284], [285]. This enables personalized strategies tailored to TME features like cellular heterogeneity, hypoxia, and pH gradients. Smart nanomaterials exemplify this trend, allowing real-time imaging, precise treatment, and guided tissue regeneration after lesion elimination [286], [287]. Theranostic digital twins (TDT) serves as a concrete application example of AI-driven deep integration of multimodal data [288], encompassing physicochemical data (RPT half-life and LET), pharmacological data (including metabolic pathways and drug interactions), physiological data (organ function and receptor density), radiobiological data (including tumor oxygenation status and radiosensitivity), and immunological data (immune cell types and cytokine expression) to construct patient-specific models (Fig. 8). For instance, before treatment, TDT is used to simulate different injection strategies (such as injection activity, frequency, and intervals) to personalize the optimal dosing plan for patients [289]. Before administration, it predicts the toxicity of radiation to risk organs (such as the kidneys, salivary glands, and bone marrow), thereby adjusting the dosage to reduce side effects [290]. These models simulate the spatiotemporal distribution of therapeutic agents within the TME and facilitate the dynamic optimization of dosing regimens for precise drug delivery. However, this technology still confronts challenges related to the standardization of multimodal data integration, the assurance of privacy protection, and the completion of clinical validation.

Furthermore, developing novel delivery systems and engineered cellular therapies is crucial. Currently, co-delivery systems for RPT and non-ICI ITs (e.g., CAR-T cells, cytokines) remain underdeveloped. Novel carriers, including radiation-responsive biomaterials or stimuli-sensitive NPs, hold promise for achieving precise spatiotemporal co-delivery, reducing side effects, and improving therapeutic sensitization [291]. The application of the CRISPR-Cas9 system for genetically editing CAR-T cells has shown that these engineered cells exhibit a radiosensitizing effect when combined with RT, a finding validated in preclinical models of leukemia, melanoma, and glioblastoma [292]. Genetically modifying CAR-T cells to secrete radiosensitizers, such as ROS-producing enzymes, or to express radiation-activated prodrug-converting enzymes, could represent a novel strategic direction for enhancing the synergy between cell therapy and RPT. Moreover, the role of therapy-induced senescence in drug resistance requires further investigation, and targeted elimination of senescent cells may prove beneficial in overcoming immunosuppressive effects and enhancing anti-tumor immune responses. Emerging strategies, including real-time molecular imaging, synthetic biology tools, and AI-optimized therapy, are poised to drive the clinical translation of these innovative approaches.

## Conclusions

The TME plays a bridging role in the synergistic enhancement of RPT and IT. Specifically, RPT reshapes the TME into an immunologically active state, thus providing targets and synergy for IT. Moreover, the TME features like vascular distribution, hypoxia, ECM density, and immunosuppressive components collectively regulate drug delivery and efficacy, while good vasculature aids drug transport. Although clinical trials of RPT combined with IT have demonstrated survival benefits, challenges remain. To overcome these challenges, future strategies should focus on remodeling the TME. Furthermore, emerging strategies and technologies, including senolytic therapies, nanodelivery systems, gene editing, AI-driven multimodal data integration, and TDT, should be integrated to enable dynamic monitoring and personalized optimization of TME-directed therapies. Ultimately, this comprehensive approach will pave new pathways toward achieving durable clinical responses with RPT-IT combinations.

## Abbreviations

BSA: Bovine serum albumin;

CAFs: Cancer-associated fibroblasts;

CAR-T: Chimeric antigen receptor T-cell;

CDNPs: Cyclodextrin nanoparticles;

CEA: Carcinoembryonic antigen;

cGAS: Cyclic GMP-AMP synthase;

CNS: Central nervous system;

CT: Computed tomography;

CTL: Cytotoxic T lymphocyte;

CTLA-4: Cytotoxic T-lymphocyte-associated protein 4;

DAMPs: Damage-associated molecular patterns;

DCs: Dendritic cells;

DNA: Deoxyribonucleic acid;

DRD2: Dopamine receptor D2;

ECM: Extracellular matrix;

EDTMP: Ethylenediamine tetramethylene phosphonate;

ERK: Extracellular signal-regulated kinase;

EVs: Extracellular vesicles;

Fas: Fas cell surface death receptor;

FMISO: Fluoromisonidazole;

GDNF: Glial cell line-derived neurotrophic factor;

GRPR: Gastrin-releasing peptide receptor;

HA: Hyaluronic acid;

HSP: Heat shock protein

HMGB1: High-mobility group box 1;

ICAM-1: Intercellular adhesion molecule 1;

ICD: Immunogenic cell death;

ICIs: Immune checkpoint inhibitors;

IFP: Interstitial fluid pressure;

IL-6: Interleukin-6;

IL-13Rα2: Interleukin-13 receptor alpha 2;

IFN: Interferon;

IT: Immunotherapy;

LET: Linear energy transfer;

mCRPC: Metastatic castration-resistant prostate cancer;

MDSCs: Myeloid-derived suppressor cells;

mGluR1: Metabotropic glutamate receptor 1;

MHC: Major histocompatibility complex;

MSLN: Mesothelin;

MUC-1: Mucin-1;

MVD: Microvascular density;

M6P: Mannose-6-phosphate;

NK: Natural killer;

NKG2D: Natural killer group 2 member D;

NP: Nanoparticle;

NSCLC: Non-small cell lung cancer;

PD-L1: Programmed cell death ligand 1;

PNI: Perineural invasion;

PSA: Prostate-specific antigen;

PSMA: Prostate-specific membrane antigen;

γH2AX: Phosphorylated histone H2AX;

ROS: Reactive oxygen species;

RPT: Radiopharmaceutical therapy;

RT: Radiotherapy;

SA-β-Gal: Senescence-associated β-galactosidase;

SASP: Senescence-associated secretory phenotype;

SAzyme: Single-atom nanozyme;

SDF-1: Stromal cell-derived factor 1;

STING: Stimulator of interferon genes;

SST2: Somatostatin receptor subtype 2;

TAM: Tumor-associated macrophages;

TDT: Theranostic digital twins;

TLR: Toll-Like receptor;

TNF-α: Tumor necrosis factor-α;

TME: Tumor microenvironment;

Tregs: Regulatory T cells;

VEGF: Vascular endothelial growth factor;

VEGFR: Vascular endothelial growth factor receptor

## Ethics approval and consent to participate

Not available.

## Funding

This work was supported by the National Health Commission of the People’s Republic of China-Zhejiang Province Jointly Constructed Project, Zhejiang Province Medical and Health Science and Technology Plan (WKJ-ZJ-2517), and the Clinical-Basic Joint Research Project of Zhejiang Provincial People’s Hospital Affiliated with Hangzhou Medical College (C-2025-YXLH09). We appreciated the support of the Chinese Scholarship Council (202206240086).

## Data Availability

Not available.
